# Exosomal *FMR1-AS1* facilitates maintaining cancer stem-like cell dynamic equilibrium via TLR7/NFκB/c-Myc signaling in female esophageal carcinoma

**DOI:** 10.1186/s12943-019-0949-7

**Published:** 2019-02-08

**Authors:** Wei Li, Liyuan Zhang, Binbin Guo, Jieqiong Deng, Siqi Wu, Fang Li, Yirong Wang, Jiachun Lu, Yifeng Zhou

**Affiliations:** 1 Department of Genetics, Medical College of Soochow University, Suzhou, 215123 China; 2 Department of Radiotherapy & Oncology, The Second Affiliated Hospital of Soochow University, San Xiang Road No. 1055, Suzhou, 215004 China; 3 The Institute for Chemical Carcinogenesis, The State Key Lab of Respiratory Disease, Guangzhou Medical University, Guangzhou, 510182 China

**Keywords:** ESCC, lncRNA, Exosome, CSC, TLR7, NFκB, c-Myc

## Abstract

**Background:**

Though esophageal cancer is three to four times more common among males than females worldwide, this type of cancer still ranks in the top incidence among women, even more than the female specific cancer types. The occurrence is currently attributed to extrinsic factors, including tobacco use and alcohol consumption. However, limited attention has been given to gender-specific intrinsic genetic factors, especially in female.

**Methods:**

We re-annotated a large cohort of microarrays on 179 ESCC patients and identified female-specific differently expressed lncRNAs. The associations between FMR1-AS1 and the risk and prognosis of ESCC were examined in 206 diagnosed patients from eastern China and validated in 188 additional patients from southern China. The effects of FMR1-AS1 on the malignant phenotypes on female ESCC cells were detected in vitro and in vivo. ChIRP-MS, reporter gene assays and EMSA were conducted to identify the interaction and regulation among *FMR1-AS1*, TLR7 and NFκB.

**Results:**

We found *FMR1-AS1* expression is exclusively altered and closely associated with the level of sXCI in female ESCC patients, and its overexpression may correlate to poor clinical outcome. ChIRP-MS data indicate that *FMR1-AS1* could be packaged into exosomes and released into tumor microenvironment. Functional studies demonstrated that *FMR1-AS1* could bind to endosomal toll-like receptor 7 (TLR7) and activate downstream TLR7-NFκB signaling, promoting the c-Myc expression, thus inducing ESCC cell proliferation, anti-apoptosis and invasion ability. Exosome incubation and co-xenograft assay indicate that FMR1-AS1 exosomes may secreted from ESCC CSCs, transferring stemness phenotypes to recipient non-CSCs in tumor microenvironment. Furthermore, we also found a correlation between the serum levels of FMR1-AS1 and the overall survival (OS) of the female ESCC patients.

**Conclusions:**

Our results highlighted exosomal *FMR1-AS1* in maintaining CSC dynamic interconversion state through the mechanism of activating TLR7-NFκB signaling, upregulating c-Myc level in recipient cells, which may be taken as an attractive target approach for advancing current precision cancer therapeutics in female patients.

**Electronic supplementary material:**

The online version of this article (10.1186/s12943-019-0949-7) contains supplementary material, which is available to authorized users.

## Background

Esophageal squamous cell carcinoma (ESCC), a predominant histologic type of esophageal carcinoma, is one of the most invasive human cancer in the world and the second most common cancer in China [1]. Though, esophageal cancer is three to four times more common among men than among women, this type of cancer still ranks in the top-6 incidence among women, even more than the female specific cancer types. Current epidemiologic studies often focus on the extrinsic environmental factors that may play a role in the observed sex disparity in ESCC incidence, such as tobacco use and alcohol consumption. However, the intrinsic molecular and genetic factors underlaying the incidence of ESCC based on gender remain largely unknown, especially in female patients. The sex-associated chromosome X carries around 2000 genes and makes up about 5% of the total DNA in women and 2.5% in men, including a large number of non-coding RNAs. LncRNAs are involved in multilevel regulation of gene expression, including transcriptional regulation by recruitment of chromatin-modifying complexes [2] and post-transcriptional regulation by interaction with miRNAs, mRNAs, or proteins [3]. LncRNAs could modulate numerous hallmarks of cancer, including proliferation [4], apoptosis [5], metastasis [6], and metabolism [7]. However, the roles of X-associated lncRNAs in female specific ESCC are unexplored.

Exosomes are generated inside multivesicular endosomes [8] and can be secreted from multiple types of cells and participate in intercellular communication by transmitting intracellular cargoes, such as proteins and nucleic acids [9]. It has been reported that numerous lncRNAs could be transferred between cancer cells, transmit signals and phenotypes via exosomes derived from human cervical and breast carcinomas [10, 11]. However, functions of exosomal lncRNAs derived from ESCC cells are still unknown. Emerging evidence revealed that the molecular cross-talking between CSCs and non-CSCs in tumour microenvironment plays a critical role in CSCs-non-CSCs dynamic equilibrium [12]. CSCs are not a static cell population with the capacity of initiating tumor through asymmetric cell division, but a cell population in highly dynamic equilibrium state, which could be maintained through the dedifferentiation of matured cancer cells [13, 14]. CSCs and non-CSCs are not in a motionless but in a dynamic equilibrium state: CSCs differentiate into non-CSCs under some circumstances, and non-CSCs could dedifferentiate into CSCs [12]. However, the cellular and molecular mechanisms of interconversion between differentiated non-CSCs and CSCs are still in the mist. Exosomes secreted by tumor cells provide a physical means to transfer a variety of intracellular molecules into the surrounding cells, where these molecules exert regulatory effects, and may serve as important molecular information carriers to communicate with CSCs, non-CSCs and other cells in tumor microenvironment [15, 16]. Tumor-cell-derived exosomes are found in all body fluids, upon contact with target cells, they can alter phenotypic and functional attributes of recipients, reprogramming them into active contributors to tumor growth, metastasis and immunosuppression [17–19]. However, the role of exosomes, specifically exosomal lncRNAs, in the reciprocal conversion between non-CSCs and CSCs was rarely investigated.

In this study, we report the investigation on the role of FMR1-AS1 in female-specific ESCC incidence and explored the potential implications for specific diagnosis and prognosis of female ESCC patients. Our results revealed the function of exosomal FMR1-AS1 in maintaining ESCC CSC dynamic interconversion and the possible mechanism of its action, which shed light on the functional exosomal lncRNAs might be taken as attractive targets for ESCC precision therapeutics and improved the understanding how CSCs and non-CSCs cell states coexist, transmit and evolve within tumors that may futher facilitate the development of more effective therapies.

## Methods

### Study subjects

Two hundred six fresh ESCC tissues and paired adjacent non-cancerous tissue samples were obtained from the patients in eastern China who underwent tylectomies at the Affiliate Hospitals of Soochow University (Suzhou). One hundred eighty-eight fresh ESCC tissues were collected from the patients at the Cancer Hospitals affiliated with Guangzhou Medical University in southern China and were used for further validation. None of the patients received anti-cancer treatment before surgery, including chemotherapy or radiotherapy. The clinical characteristics of the patients are listed in Additional file 1: Table S1.

### Comprehensive identification of RNA-binding proteins by mass spectrometry (ChIRP-MS)

10–20 15 cm dishes of cells were used per ChIRP-MS experiment (100million - 500million cells depending on the cell type). Cell harvesting, lysis, disruption, and ChIRP were essentially performed as previously described [20] and follow the manufacturer’s instruction (Magna ChIRP, Millipore, USA). Final protein samples were size-separated in bis-tris SDS-PAGE gels (Invitrogen) for western blots and MS. The following antibodies were used in the followed western-blotting: anti-TLR7 (ab45371, Abcam), anti-hnRNPK (ab39975, Abcam), anti-PRDX1 (ab41906, Abcam), anti-PRDX2 (ab109367, Abcam), anti-ECM1 (ab126629, Abcam) and anti-β-actin (ab8227, Abcam). See Additional file 2: Materials and Methods for details.

### Statistical analysis

The accession number for the microarray data analyzed in this paper is Gene Expression Omnibus database GEO: GSE53625 and GSE70817. The ribosome profiling data were obtained from the GSE61742. The data analysis was performed using SPSS 20.0 (IBM, US). Kaplan-Meier estimate with log-rank test was used to compare patient’s survival by different *FMR1-AS1* expression levels. For functional analysis, results were presented as mean ± SEM. Comparison of mean between two groups was conducted using Student’s t-test, while the comparison for more than two groups was conducted using one-way ANOVA. Data in abnormal distribution were analyzed by non-parametric test. Statistical significance was two-tailed and set at *P* < 0.05.

## Results

### *FMR1-AS1* highly expressed in ESCC tissues and indicate a poor prognosis in female patients

**Fig. 1:**
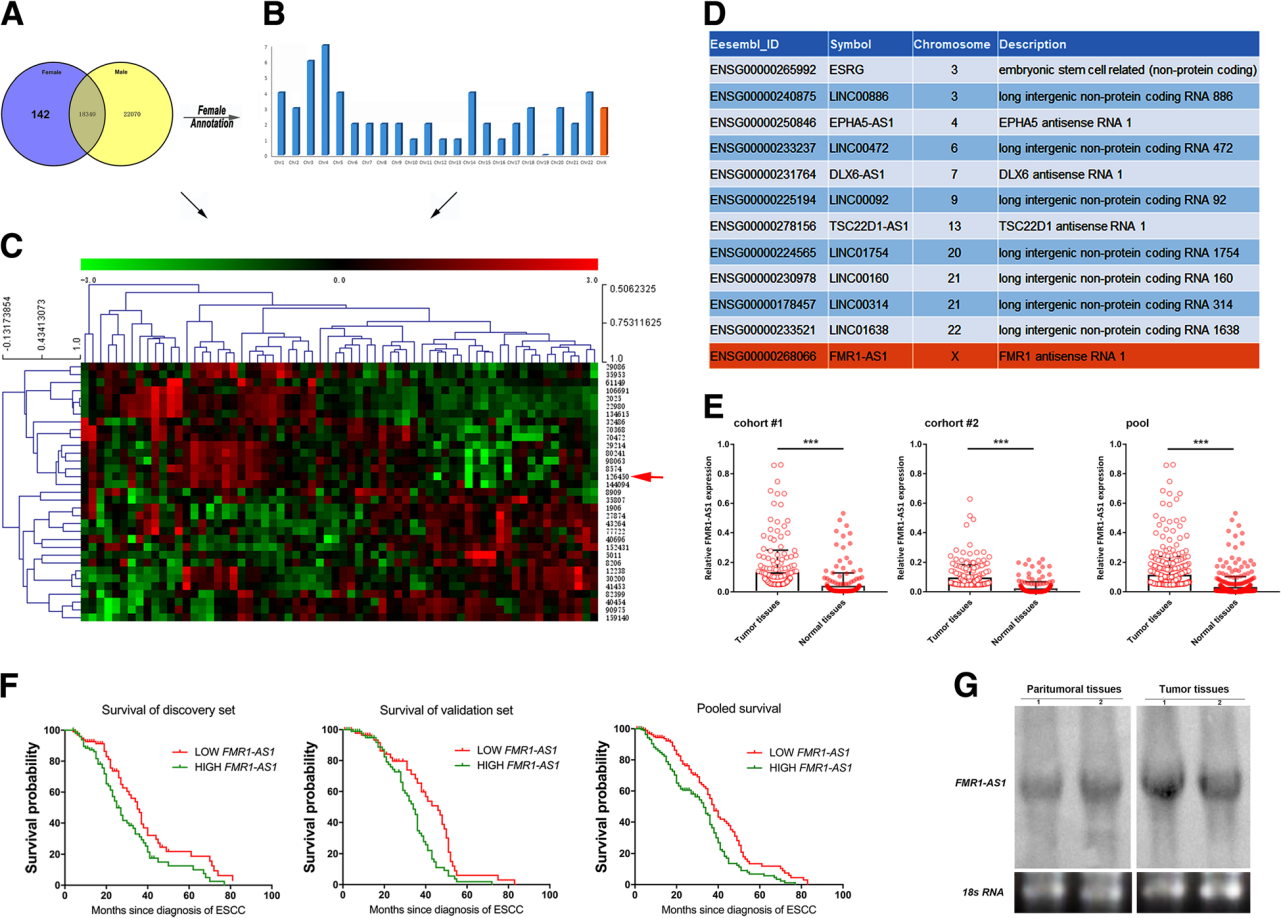
Female specific X-associated lncRNA screening and *FMR1-AS1* expression patterns in female ESCC samples and cells. **a** The venn diagram in (A) depicts the number of gene probes that are differentially expressed in the female ESCC group versus male. **b** The distribution of those female differentially expressed genes on each chromosome after annotation. **c** The heat map shows all 142 differentially expressed genes (*p* < 0.001) specific in female ESCC microarray samples, red = upregulated genes; green = downregulated genes. **d** LncRNAs that differentially expressed in female ESCC microarray samples. **e** *FMR1-AS1* expression in female ESCC and matched non-tumor tissues from Suzhou (*n* = 206) and Guangzhou (*n* = 188) (***p < 0.001). **f** Kaplan-Meier plots for the disease-free survival rate of female ESCC patients in groups of *FMR1-AS1* high or low expression levels in the Suzhou cohort (n = 206, discovery set), Guangzhou cohort (n = 188, validation set), and pooled populations (*n* = 394, pooled analysis). **g** Northern-blot of *FMR1-AS1* in two pairs of ESCC tissue samples

We first compared the lncRNA expression profiles of 179 pairs ESCC tissues and its adjacent normal tissues. Unsupervised hierarchical clustering was used to divide the ESCC tissues into female and male groups. In total, 40,410 differently expressed probes with adjusted *P* < 0.01 were selected from the male group (146 patients), and 18,482 differently expressed probes were selected from the female group (33 patients, adj. *P* < 0.01). One hundred forty two probes differently expressed in the female ESCC patients were not included in those of male patients (Fig. 1a-c). Among these 142 differently expressed genes, we identified 12 lncRNAs (Fig. 1d). The only X chromosome lncRNA, FMR1-AS1, comes into our notice. In our patient samples expression measurement, *FMR1-AS1* was significantly higher (~ 2.65-fold, *P* < 0.001) in the tumor tissues than in the adjacent non-neoplastic tissues in female patients from Suzhou (*n* = 206). Consistent with Suzhou samples, the *FMR1-AS1* level was also notably higher (~ 2.3-fold, *P* < 0.001) in female tumor tissues than in adjacent non-neoplastic tissues from Guangzhou (*n* = 188) (Fig. 1e). However, no significant differences found in male ESCC samples from both area (Suzhou: 192, Guangzhou: 168) (Additional file 3: Figure S1a-c and Additional file 4).

Next, we determined the correlation between the expression levels of *FMR1-AS1* and the overall survival (OS) of the female ESCC patients. The ESCC patients were classified into high and low *FMR1-AS1* groups, according to the medium expression level of *FMR1-AS1* among female ESCC tissues. A log-rank test and Kaplan-Meier survival curves in the discovery, validation and the pooled sets were used to compare the two *FMR1-AS1* groups. We found that female patients from the discovery set (Suzhou: 206) in the high *FMR1-AS1* subgroup had a lower OS than those in the low *FMR1-AS1* subgroup (HR = 1.618; 95%CI = 1.117–2.345; *P* = 0.009). And this result was confirmed in the validation set (Guangzhou: 188, HR = 1.768; 95%CI = 1.189–2.631; *P* = 0.0031) and the pooled set (HR = 1.679; 95%CI = 1.28–2.202; *P* = 0.0001), the high *FMR1-AS1* group showed a lower OS of female ESCC patients (Fig. 1f, Additional file 3: Figure S1d and Additional file 4). The sequence of full-length *FMR1-AS1* has been documented in previous studies that use rapid amplification of cDNA ends (RACE) [21]. We also used northern blot to verify the expected size of *FMR1-AS1* in the total RNA of two pairs of human ESCC tissue samples (Fig. 1g).

### *FMR1-AS1* transcriptionally regulated by NFκB and associated with skewed X-chromosome inactivation in female ESCC patients

**Fig. 2:**
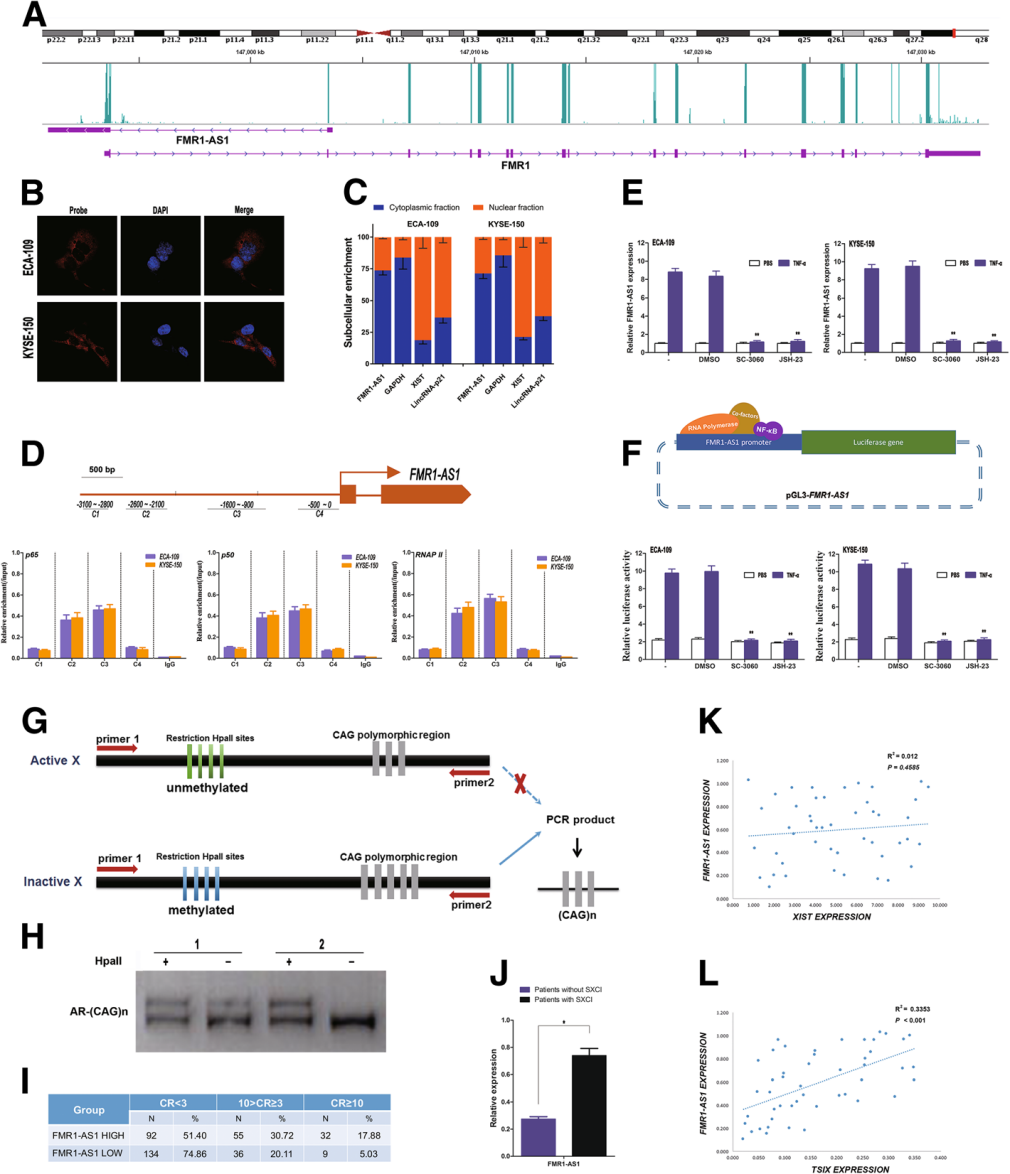
Biological characterization of *FMR1-AS1*. **a** Ribosome occupancy at the *FMR1* and *FMR1-AS1* locus. The green peaks indicate reads density that mapped at the region. **b** RNA fluorescence in situ hybridization to localize *FMR1-AS1*. **c** Relative abundance of *FMR1-AS1* transcript in cytoplasm, nucleus and chromatin in female ESCC cells. *GAPDH*, *XIST* and *LincRNA-p21* were used as controls respectively. **d** Expression of *FMR1-AS1* in ESCC cells induced by TNF-α with or without NF-kB inhibition by sc-3060 or JSH-23 (mean ± SD, *n* = 5, *p < 0.01). **e** Chromatin immunoprecipitation showing p65, p50 and RNAP II occupancy at the *FMR1-AS1* locus in ESCC cells. Locations of amplicons (C1–C4) are indicated in the scheme above. Values represent the enrichment of bound protein fractions relative to input (mean ± SD, *n* = 3). **f** Luciferase reporter assay in ESCC cells induced by TNF-α with or without NF-kB inhibition by sc-3060 or JSH-23, and the reporter constructs expressing the luciferase gene under *FMR1-AS1* promoter segment (mean ± SD, n = 5, *p < 0.01). **g**, **h** sXCI detection assays, based on *HpaII* digested genomic DNA PCR on the highly polymorphic CAG repeat-region of androgen receptor (*AR*), which amplifies the undigested inactive X chromosome only. **i** sXCI frequency in the *FMR1-AS1* high/low groups of female ESCC tissues (*P*_χ_^2^ < 0.001). **j** *FMR1-AS1* expression in female ESCC patients with or without sXCI (CR ≥ 3). **k**, **l** Correlation analysis on the expression level of *FMR1-AS1, XIST* (R^2^ = 0.012, *P* < 0.4585) and *TSIX* (R^2^ = 0.3353, *P* < 0.001)

To further verify the coding potential of *FMR1-AS1*, we re-analyzed a dataset of human lymphoblastoid cell ribosome sequencing profiling and measured the ribosome occupancy level at the *FMR1* gene locus. As expected, the ribosome profiling reads are highly concentrated within the coding region of *FMR1* gene rather than *FMR1-AS1* (Fig. 2a). In addition, the PhyloCSF score is − 101.3062, lower than the cutoff 60.7876, which further supports the finding that *FMR1-AS1* has no protein-coding potential. Confocal microscopy for fluorescent in situ hybridization (FISH) showed that *FMR1-AS1* located primarily in the cytoplasm (Fig. 2b), which was confirmed by qPCR in nuclear/cytoplasm fractionation (Fig. 2c), may exert its biological function in the cytoplasm of ESCC cells.

To investigate upstream regulation on *FMR1-AS1*, we searched the TFBS motifs in FMR1-AS1 promotor region through TRANSFAC and JASPAR matrix using LASAGNA-Search 2.0. Interestingly, we found several evolutionarily conserved NFκB-binding motifs within the promoter of *FMR1-AS1*(Additional file 5: Figure S2a and Additional file 4). As core factors of NFκB with transcription activity, p50 and p65 showed significant increases at the specific sites within the promoter of *FMR1-AS1*, while no enrichment was shown at the negative control sites that contain irrelevant regions (Fig. 2d). Sc-3060 and JSH-23, two inhibitors for NFκB nuclear translocation, abrogated the TNF-α-induced *FMR1-AS1* upregulation in female ESCC cells (Fig. 2e, Additional file 5: Figure S2b and Additional file 4). Next, we cloned the proximal promoter region of *FMR1-AS1* to construct reporter plasmids (pGL3-*FMR1-AS1*) and performed luciferase assays in ESCC cells or cells treated with two inhibitors of NFκB, Sc-3060 and JSH-23. As illustrated in Fig. 2f, compared to the control cells, the NFκB-inhibited cells had a significantly lower luciferase activity.

*FMR1-AS1* is antisense to the CGG repeat region of the *FMR1* gene in the same locus and have been reported to be silenced by the repeat expansion [22]. This CGG repeat length of *FMR1* locus have been reported a role in determining X-chromosome inactivation [23]. While sXCI also have been considered as a predisposing factor for the early development of esophageal carcinoma [24], it is biologically reasonable to expect a possible relationship between the female ESCC sXCI and the *FMR1-AS1* expression.

Both CR ≥ 3 and CR ≥ 10 were used as the criteria of sXCI in the subjects (Fig. 2g, Additional file 5: Figure S2c and Additional file 4). In total of 394 ESCC samples, the heterozygosity frequency of AR is about 90.86% and 358 ESCC patients were used to detect sXCI. Interestingly, more sXCI patients were found in the high *FMR1-AS1* expression group than in the low *FMR1-AS1* expression group (CR ≥ 3: 48.60% versus 25.14%; CR ≥ 10: 17.88% versus 5.03%) (Fig. 2h, i). Furthermore, the sXCI patients showed a significantly higher expression level of *FMR1-AS1* than the non-sXCI patients (Fig. 2j).

For females, most of expression of X-linked genes are regulated by XCI, which occurs during fetal period and determined by *XIST* and its antisense, *TSIX*. Thus, we measured the expression levels of *XIST*, *TSIX* and *FMR1-AS1* in ESCC tissues to determine whether *FMR1-AS1* had a correlation with *XIST* or *TSIX*. The results suggesting that the *TSIX* expression may also influence *FMR1-AS1* in female ESCC (Fig. 2k, l).

### Effects of ectopic *FMR1-AS1* expression on ESCC cell malignant phenotypes

**Fig. 3:**
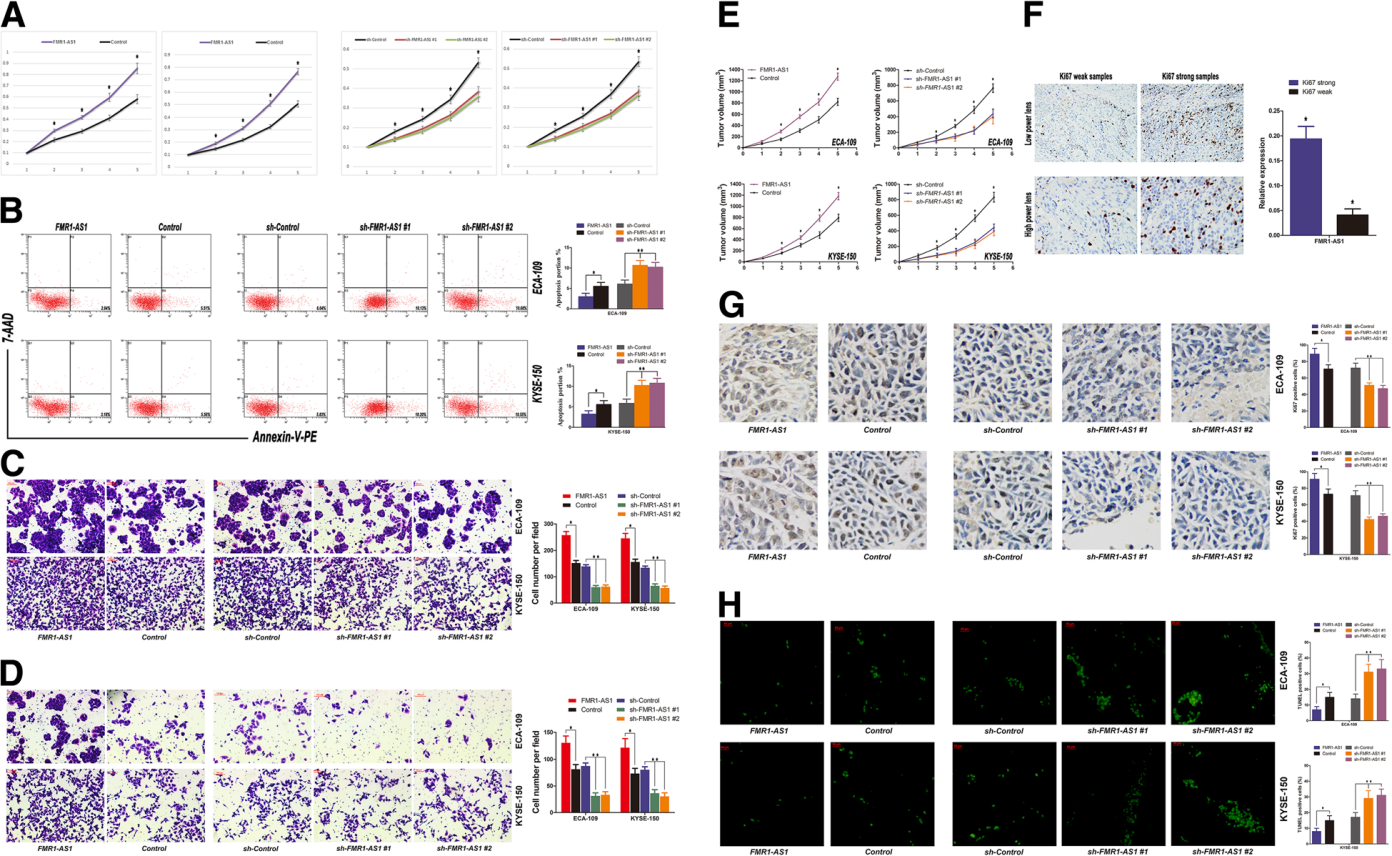
Effects of ectopic *FMR1-AS1* expression on female ESCC cells. **a** Cell proliferation assay on *FMR1-AS1-*upregulated, *FMR1-AS1-*downregulated and respective control ESCC cells using the Cell Counting Kit-8 (mean ± SD, *n* = 6, *p < 0.05). **b** Apoptosis rates on ESCC cells transfected with *FMR1-AS1*, Control, sh-Control, sh-*FMR1-AS1* #1 and sh-*FMR1-AS1* #2 lentiviruses, using flow cytometry (mean ± SD, n = 6, *p < 0.05). **c**, **d** Migration and invasion of ESCC cells transfected with *FMR1-AS1*, Control, sh-Control, sh-*FMR1-AS1* #1 and sh-*FMR1-AS1* #2 lentiviruses. Right panel is the quantification of migrated/invaded cells (mean ± SD, n = 6, *p < 0.05). **e** Xenograft mice subcutaneously implanted *FMR1-AS1-*upregulated, *FMR1-AS1-*downregulated and respective control ESCC cells (mean ± SD, n = 6, *p < 0.05). **f** Ki67 immunostaining in female ESCC samples and *FMR1-AS1* expression in Ki67 strong and weak samples. **g** Ki67 immunostaining in xenograft tumors of *FMR1-AS1-*upregulated, *FMR1-AS1-*downregulated and respective control ESCC cells. **h** TUNEL staining in xenograft tumors of *FMR1-AS1-*upregulated, *FMR1-AS1-*downregulated and respective control ESCC cells

We next examined the effects of *FMR1-AS1* on female ESCC cell phenotypes (Additional file 6: Figure S3a and Additional file 4). The results showed that overexpression of *FMR1-AS1* in ECA-109 and KYSE-150 substantially promoted cell proliferation, whereas the knockdown of *FMR1-AS1* significantly abolished this effect (Fig. 3a). We also found that *FMR1-AS1* overexpression resulted in a significant accumulation of ECA-109 and KYSE-150 cells in the G2-M phase using PI-staining and FACS sorting. No significant effects on other cell phase percentages were observed (Additional file 6: Figure S3b and Additional file 4). Interestingly, knockdown of *FMR1-AS1* significantly increased cell apoptosis of ECA-109 and KYSE-150, while overexpression of *FMR1-AS1* caused a modest but significant decrease of apoptosis (Fig. 3b, Additional file 6: Figure S3c and Additional file 4). We then conducted transwell and wound-healing assays, using ESCC cells with *FMR1-AS1* overexpressed and knocked-down. Both transwell and wound-healing assays demonstrated that the migration capability of ECA-109 and KYSE-150 cells was significantly induced by *FMR1-AS1* overexpression and reduced by *FMR1-AS1* knockdown (Fig. 3c, d, Additional file 6: Figure S3d and Additional file 4). We also used ECA-109 and KYSE-150 cells to perform a colony formation assay and found that the overexpression of *FMR1-AS1* markedly increased the colony formation ability of ESCC cells (Additional file 6: Figure S3e, f and Additional file 4).

To probe the effects of *FMR1-AS1* on cancer cell dynamics in vivo, we injected *FMR1-AS1*-upregulated cells, *FMR1-AS1*-downregulated cells and control cells into the hind flanks of nude mice. We observed that compared with control xenografts, the growth of the *FMR1-AS1*-upregulated xenografts was significantly promoted, whereas the growth of the *FMR1-AS1*-knockdown xenografts was substantially decreased (Fig. 3e). We also categorized 32 ESCC tissues according to the Ki67 expression (≥30%, strong staining; < 30%, weak staining) and found that the expression of *FMR1-AS1* was much higher in the group of Ki67 strong staining than the group of weak Ki67 (Fig. 3f). Furthermore, Ki67 immunostaining also showed more proliferation in xenograft tumors of the *FMR1-AS1*-upregulated ESCC cells, while TUNEL staining showed more apoptosis in *FMR1-AS1*-downregulated ESCC xenograft (Fig. 3g, h).

### Exosome transmitted *FMR1-AS1* promotes c-Myc expression through interacting with TLR7 and activating NFκB signaling

**Fig. 4:**
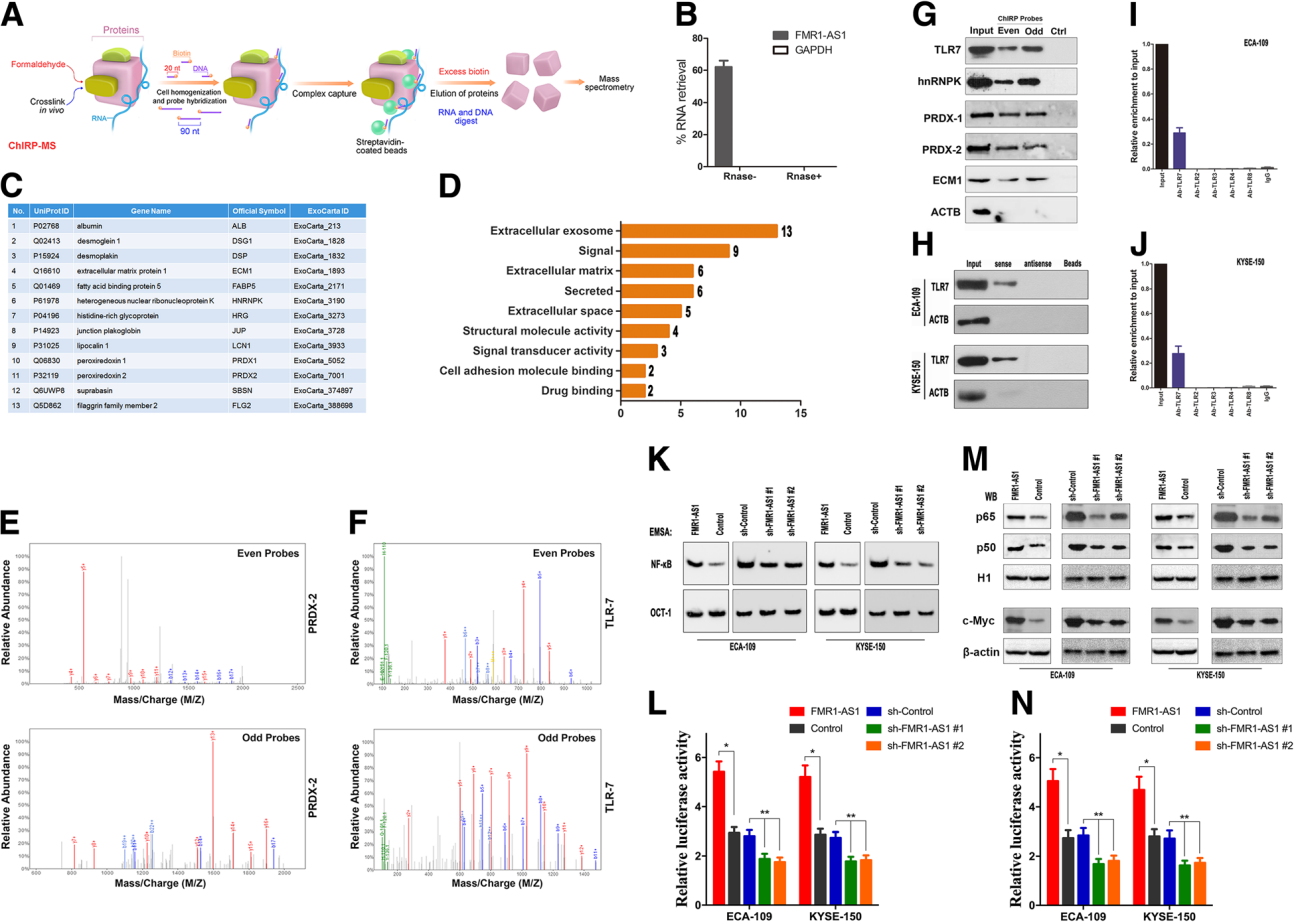
*FMR1-AS1* could be packaged into exosomes and activates TLR7- NFκB-c-Myc signaling. **a** Outline of the ChIRP-MS workflow. Briefly, cells are crosslinked by 3% formaldehyde for 30 min and solubilized by sonication. *FMR1-AS1* are pulled out by biotinylated antisense oligos, and associated proteins are eluted with free biotin. Each size fraction is subjected to LC/MS-MS identification. **b** Over 60% of *FMR1-AS1* was retrieved from the cell by ChIRP, while no Gapdh was detected. RNase treatment eliminates *FMR1-AS1* transcripts prior to pull-down. **c** *FMR1-AS1* associated proteins that were identified as exosomal proteins by ExoCarta database. **d** Functional classification of *FMR1-AS1* ChIRP-retrived proteins. **e**, **f** Identification of PRDX2 and TLR7 in both even/odd probe group retrieved proteins using Mass spectrometry. **g** Validation of ChIRP-enriched proteins by immunoblotting using TLR7, hnRNPK, PRDX1, PRDX2, ECM1 and β-actin antibodies. **h** Western-blotting validation of TLR7 in *FMR1-AS1* pulldown protein extractions. **i**, **j** RNA-immunoprecipitation (RIP) experiments were performed using TLR2/3/4/7/8 and IgG antibodies to immunoprecipitate and a primer to detect *FMR1-AS1* in ECA-109 and KYSE-150 cells. **k** NFκB activities in *FMR1-AS1-*upregulated, *FMR1-AS1-*downregulated and respective control ESCC cells, examined by EMSA. **l** NFκB activity *FMR1-AS1-*upregulated, *FMR1-AS1-*downregulated and respective control ESCC cells, examined by luciferase reporter plasmid with *FMR1-AS1* promoter (mean ± SD, n = 6, *p < 0.05). **m** Western blotting showing nuclear p65, p50 and c-Myc levels in *FMR1-AS1-*upregulated, *FMR1-AS1-*downregulated and respective control ESCC cells. **n** NFκB activity in *FMR1-AS1-*upregulated, *FMR1-AS1-*downregulated and respective control ESCC cells, examined by luciferase reporter plasmid with *MYC* gene promoter (mean ± SD, n = 6, *p < 0.05)

To search for the potential interacting molecules of *FMR1-AS1* to regulate target genes at distal genomic loci, we purified endogenous *FMR1-AS1* complexes using modified ChIRP [25] that allows unbiased high-throughput discovery of *FMR1-AS1* associated proteins (Fig. 4a). We designed 16 probes (even/odd group) against human *FMR1-AS1* RNA (Additional file 7: Table S2). In female ESCC cell line (ECA-109), we confirmed that *FMR1-AS1* was completely solubilized by sonication and that over 75% of *FMR1-AS1* RNA was selectively retrieved without enrichment of housekeeping GAPDH mRNA (Fig. 4b). There are 24 proteins both even and odd probes retrieved, among which 13 proteins are recorded as extracellular exosome components in DAVID and Exocarta database (Fig. 4c, d, Additional file 8: Table S3), indicating *FMR1-AS1* which may be specifically sorted into exosomes. Specific *FMR1-AS1* retrieved proteins identified by LC-MS/MS (Fig. 4e, f) was validated by ChIRP-western (Fig. 4g).

Among all the ChIRP-retrieved proteins, Toll-like receptor 7 (TLR7) came into notice. TLR7 is a pattern-specific immune receptor and localizes to the endosomal compartment. It is activated by antiviral compounds, viral RNA, single-stranded oligoribonucleotides and short interfering RNAs [26–28]. Further consistent with *FMR1-AS1* ChIRP-MS data, a strong signal for TLR7 could be detected in the proteins that were pulled-down with the sense strand of *FMR1-AS1* (Fig. 4h). To further confirm this interaction, we examined this interaction between *FMR1-AS1* and TLR7 by RNA immunoprecipitation (RNA-IP). The results showed a significant enrichment of *FMR1-AS1* bound to TLR7, compared with the non-specific IgG control (Fig. 4i, j). Taken together, these results demonstrated a specific association between TLR7 and *FMR1-AS1*.

Exosomes are lipid-bilayer vesicles containing diverse proteins, RNAs, and DNAs [29, 30]. These contents have been shown to be recognized by multiple PRRs, including TLR7 [31–33]. However, the ability to stimulate TLR7 depended on GU-rich elements rather than on the exact GUUGUGU motif, as the motif analysis of previous studies have defined specific GU-rich 4-mer sequences like UUGU, GUUC, GUUU, UUUC, UGUU, or UCUC activating human TLR7 [34–36]. As expected, *FMR1-AS1* contains 10 UUGU, 8 GUUC, 15 GUUU, 17 UUUC and 16 UGUU sites of these motif effectively engaged with TLR7.

In order to identify the associated biological processes and the corresponding signaling pathways of *FMR1-AS1*, we applied the gene set enrichment analysis (GSEA) to identify *FMR1-AS1* associated gene sets and molecular signatures on the gene expression profiles (GSE70817) with *FMR1-AS1* overexpressed and silenced [37]. The gene sets with significantly different levels of expression (*P* < 0.005) were picked up for GSEA. Interestingly, GSEA indicated that several classic stemness-associated signaling pathways, such as Wnt and Notch signaling, were significantly enriched in the cells with aberrantly expressed *FMR1-AS1* (Additional file 9: Figure S4a and Additional file 4). The TOP-FLASH reporter and western-blotting suggested exogenous *FMR1-AS1* in the ESCC cells induced Wnt and Notch signaling, but silencing *FMR1-AS1* abolished this ability (Additional file 9: Figure S4b, c and Additional file 4). Moreover, GSEA results also indicated four putative NFκB associated gene signatures that were significantly enriched in both *FMR1-AS1* overexpressed and knocked-down cells (Additional file 9: Figure S3d and Additional file 4). The EMSA, luciferase reporter assays and western-blotting demonstrated that NFκB activity in ESCC cells was significantly increased by exogenous *FMR1-AS1* and suppressed upon *FMR1-AS1* silencing (Fig. 4k-m), strongly suggested that *FMR1-AS1* is involved in NFκB activation. Among those Wnt and Notch signaling target genes, c-Myc is a well-known cancer stem factor, which also is under direct transcription regulation of NFκB [38]. Reasonably, c-Myc level was also increased by exogenous *FMR1-AS1* and suppressed upon *FMR1-AS1* silencing (Fig. 4m, n). Our results of luciferase reporter assay with inhibition of NFκB also confirmed the transcription regulation role of NFκB on c-Myc in human ESCC (Additional file 9: Figure S4e and Additional file 4). Additionally, MYC also exhibit overexpression status in female ESCC patients based on TCGA and our sample data (Additional file 9: Figure S4f, g and Additional file 4).

### Intercellular transfer of *FMR1-AS1* by exosomes disseminates ESCC stemness phenotypes

**Fig. 5:**
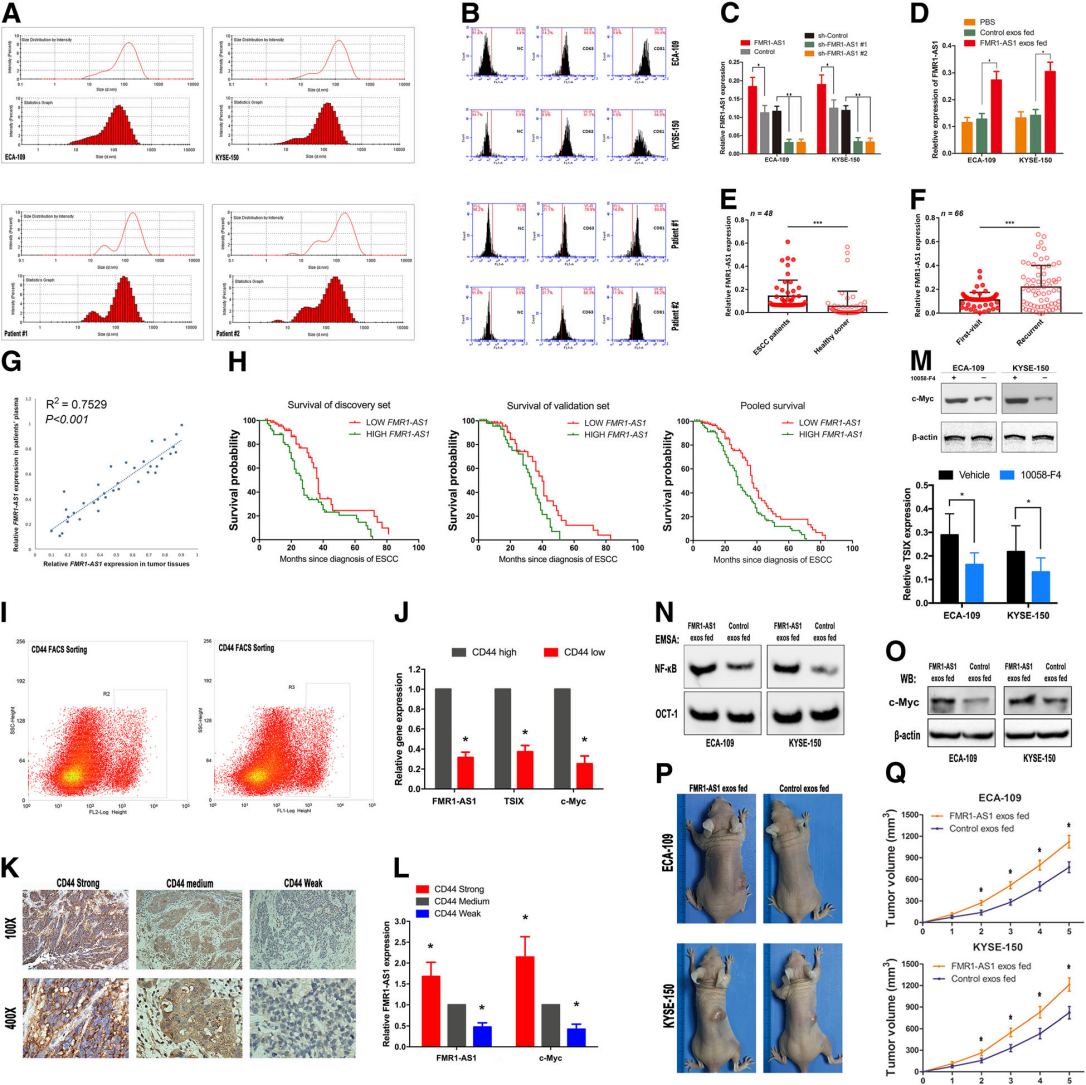
Intercellular transfer of *FMR1-AS1* by exosomes disseminates ESCC stem-like phenotypes. **a** Size distributions and statistics graph of exosomes isolated from CM of ECA-109 and KYSE-150 cells (Upper) and serum of female ESCC patients (Lower). **b** FACS analysis of exosomes isolated from the CM of ECA-109 and KYSE-150 cells (Upper) and serum of female ESCC patients using CD63 and CD81(Lower). **c** qRT-PCR analysis of *FMR1-AS1* in exosomes isolated from the CM of *FMR1-AS1-*upregulated, *FMR1-AS1-*downregulated and respective control ESCC cells (n = 3). **d** qRT-PCR analysis of *FMR1-AS1* in ESCC cells after 48 h incubation with indicated exosomes (PBS as control, n = 3). **e** qRT-PCR analysis of *FMR1-AS1* in serum exosomes from female ESCC patients (*n* = 48, ***P < 0.001), compared with that from healthy control. **f** qRT-PCR analysis of *FMR1-AS1* in serum exosomes from female ESCC patients (first visit patients group versus recurrent patients group, *n* = 66, ***P < 0.001). **g** The consistency between *FMR1-AS1* tumor expression and serum expression within individual ESCC patients (R^2^ = 0.7529, *P* < 0.001). **h** Kaplan-Meier plots for the disease-free survival rate of female ESCC patients in groups of serum *FMR1-AS1* high or low expression levels in the Suzhou cohort (*n* = 146, discovery set), Guangzhou cohort (*n* = 107, validation set), and the overall populations (*n* = 253, pooled analysis). **i**, **j** Flow cytometry assay using antibodies against CD44 cell surface markers to obtain two subtypes of tumor cells (CD44 high and CD44 low) from 12 female ESCC patients. The histograms showing expression levels of *FMR1-AS1*, *c-Myc* and *TSIX* in these two cell subtypes (*P < 0.05). **k**, **l** CD44 immunostaining in female ESCC samples and *FMR1-AS1,* c*-Myc* expression levels in CD44 strong, medium and weak samples (*P < 0.05). **m** Western blotting of the c-Myc protein level and *TSIX* expression level on ESCC cells treated with or without c-Myc inhibitor (*P < 0.05). **n** NFκB activities of ESCC cells after 48 h incubation with indicated exosomes, examined by EMSA. **o** Western blotting showing c-Myc levels in ESCC cells after 48 h incubation with indicated exosomes. **p**, **q** Subcutaneous xenograft assay of *FMR1-AS1*-upregulated, *FMR1-AS1*-downregulated and respective control ESCC cells in nude mice with intratumoral injection of indicated exosomes (*n* = 5 per group)

We next investigated the existing pattern of exosomal *FMR1-AS1*. The levels of *FMR1-AS1* in culture medium (CM) of the ESCC cells remained unchanged upon RNase treatment, but they were significantly decreased when treated with RNase and Triton X-100 simultaneously (Additional file 10: Figure S5a and Additional file 4). The result indicated that extracellular *FMR1-AS1* was mainly wrapped by membrane instead of being directly released. The data from exoRbase also indicate *FMR1-AS1* could be detected in human blood exosomes (Additional file 10: Figure S5b and Additional file 4). We then purified exosomes from ESCC cell CM and patients’ blood serum and confirmed their existence by typical particle size and exosome markers CD63 and CD81 (Fig. 5a, b). As expected, the exosomal *FMR1-AS1* levels were significantly higher in the *FMR1-AS1* upregulated cells and were lower in the *FMR1-AS1* downregulated cells than in the corresponding control cells respectively (Fig. 5c).

Cell-secreted exosomes and capped cargoes can be internalized by neighboring cells [30]. As expected, the intracellular levels of *FMR1-AS1* were increased upon incubation with exosomes from the *FMR1-AS1* overexpressed cells, but not the *FMR1-AS1* knocked-down cells (Fig. 5d). Intriguingly, serum exosomal *FMR1-AS1* expression levels were significantly higher in the female ESCC patients than in the healthy control subjects (Fig. 5e), as well as higher in ESCC recurrent patient group than in first visit group (Fig. 5f). We observed the consistency between *FMR1-AS1* tumor expression and serum expression within individual ESCC patients (Fig. 5g). Moreover, we also determined the correlation between the serum levels of *FMR1-AS1* and the overall survival (OS) of the female ESCC patients. We found that female patients from the discovery set (Suzhou: 146) in the high serum *FMR1-AS1* subgroup had a lower OS than those in the low *FMR1-AS1* subgroup (HR = 1.942; 95%CI = 1.246–3.027; *P* = 0.0029). And this result was confirmed in the validation set (Guangzhou: 107, HR = 1.784; 95%CI = 1.06–3.004; *P* = 0.0163) and the pooled set (HR = 1.761; 95%CI = 1.259–2.462; *P* = 0.0006), the high serum *FMR1-AS1* group showed a lower OS of female ESCC patients (Fig. 5h). These results indicate a potential application of *FMR1-AS1* as a biomarker of female ESCC.

The findings above rise the question that from where *FMR1-AS1* exosomes derived. Since NFκB affected Wnt and Notch pathways play crucial roles in cancer stemness, we hypothesis that *FMR1-AS1* exosomes derived from ESCC cancer stem-like cells. To address this hypothesis, we newly enrolled 12 female ESCC samples and used FACS to isolate the stem-like subpopulation with cell-surface marker CD44. Interestingly, the expression levels of *FMR1-AS1*, *TSIX* and c-Myc were significantly higher in the CD44^+^ (stem-like) ESCC subpopulation cells than in the CD44^−^ cells (Fig. 5i, j). Consistently, we categorized 32 ESCC tissues by the level of CD44 expression (strong≥50%; 50% > medium≥20%; weak< 20%) and *FMR1-AS1* and c-Myc were both observed much higher expression in the CD44 strong staining tissues than in the CD44 medium and weak samples (Fig. 5k, l).

Since the acquisition of pluripotency always accompanied by the regulation of *TSIX*, and c-Myc was found to be one of the activators of *TSIX* [39]. We then investigated the regulation role of c-Myc on *TSIX* in ESCC cells using c-Myc inhibitor. The results showed that c-Myc could also associated with *TSIX* promoter and activate *TSIX* expression in ESCC cells (Fig. 5m).

To further confirm that *FMR1-AS1* could be transferred to recipient cells via exosomes, conferring functions and phenotypes to recipient ESCC cells, we first determined the NFκB transcriptional activity in ESCC cells incubated directly with exosomes from the *FMR1-AS1* overexpressed cells. The results of luciferase reporter assay and EMSA showed that NFκB transcriptional activity was significantly increased after incubation with exosomes from the *FMR1-AS1* overexpressed cells (Fig. 5n, Additional file 10: Figure S5c and Additional file 4). And qPCR and western-blotting upon exosome incubation showed that, accompanied by NFκB activation, c-Myc level was also upregulated (Fig. 5o, Additional file 10: Figure S5d and Additional file 4). Then to demonstrate the effect of exosomal *FMR1-AS1* on ESCC phenotypes in vivo, we administered exosomes derived from the *FMR1-AS1* overexpressed and control cells intratumorally into ECA-109 and KYSE-150 xenografts. Exosomes derived from the *FMR1-AS1* overexpressed cells significantly induced the growth of ESCC xenografts in female mice (Fig. 5p, q). Furthermore, Wnt and Notch signaling were also induced after incubation with exosomes from the *FMR1-AS1* overexpressed cells, compared to exosomes from the control cells (Additional file 10: Figure S5e, f and Additional file 4). Collectively, these findings demonstrated that exosomes from the *FMR1-AS1* overexpressed cells could endow the basal cells with stem-like phenotypes via intercellular transfer of *FMR1-AS1*, through activating NFκB-dependent pathways, thus contributing to ESCC intratumorally stemness dynamic transition.

### TLR7-NFκB-c-Myc signaling pathway activation is responsible for *FMR1-AS1*-mediated reprogramming of ESCC cells

**Fig. 6:**
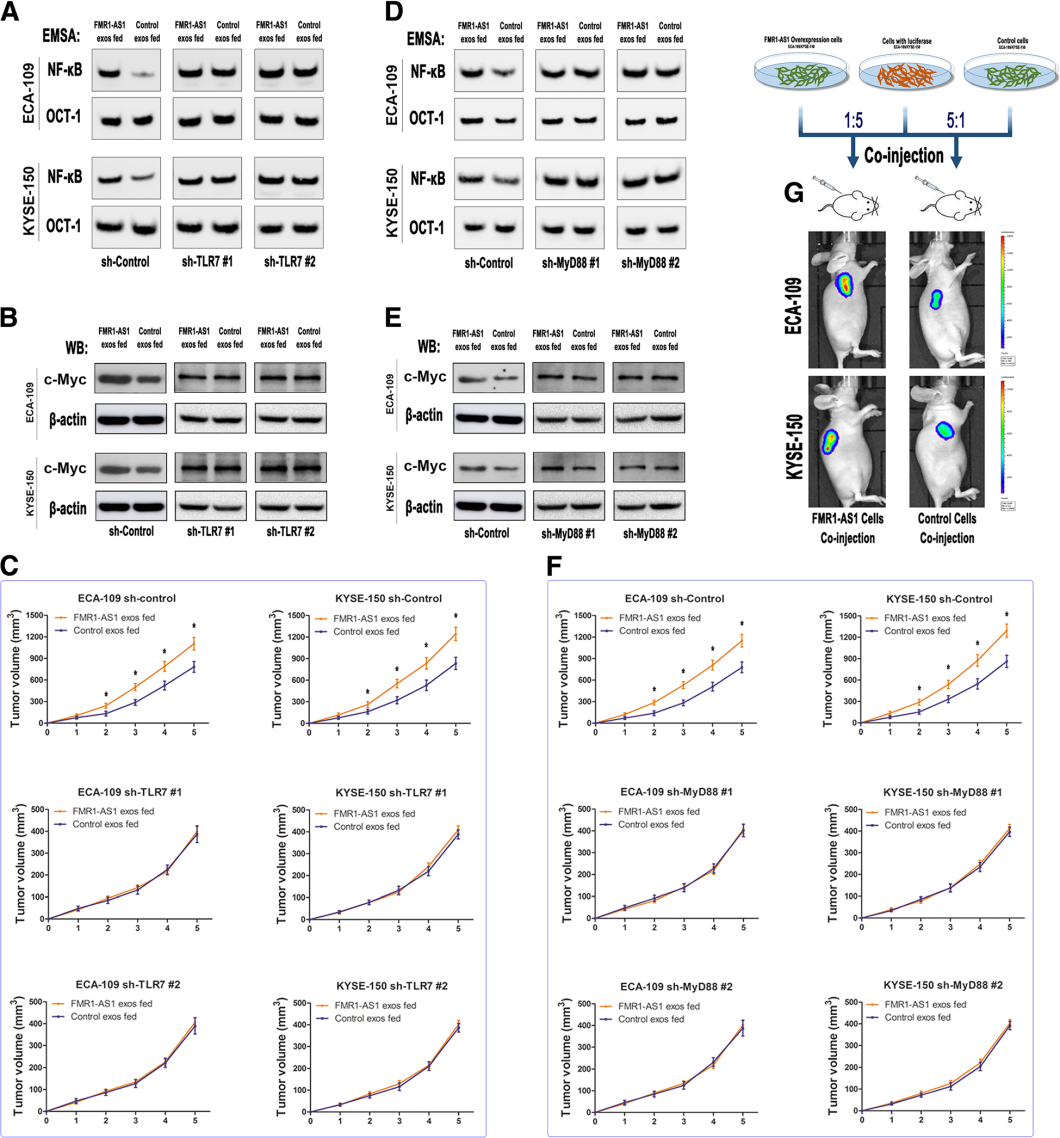
TLR7-NFκB signaling pathway activation is responsible for *FMR1-AS1*-mediated cancer stem cell transition. **a**, **b** NFκB activities and c-Myc level of ESCC cells with TLR7 knockdown after 48 h incubation with indicated exosomes, examined by EMSA and western blotting. **c** Subcutaneous xenograft assay of TLR7-downregulated and respective control ESCC cells in nude mice with intratumoral injection of indicated exosomes (n = 5 per group). **d**, **e** NFκB activities and c-Myc level of ESCC cells with MyD88 knockdown after 48 h incubation with indicated exosomes, examined by EMSA and western blotting. **f** Subcutaneous xenograft assay of MyD88-downregulated and respective control ESCC cells in nude mice with intratumoral injection of indicated exosomes (n = 5 per group). **g** Subcutaneous xenograft assay co-injected with cell mixture of ECA-109_Luc and ECA-109_*FMR1-AS1*/ECA-109_Control cells (1:5), KYSE-150_Luc and KYS-150_*FMR1-AS1*/KYSE-150_Control cells (1:5)

We further sought to identify the underlying mechanism of *FMR1-AS*-mediated transition of ESCC stem-like phenotypes. Inspired by the results above, we focused on TLR7-MyD88-NFκB signaling pathway. Therefore, by stably silencing TLR7 in ESCC cells, we found that the inhibition of TLR7 could block the activation of NFκB-c-Myc signaling pathway and suppressed the xenografts growth phenotypes by *FMR1-AS1* exosome incubation (Fig. 6a-c, Additional file 11: Figure S6a and Additional file 4). Moreover, the silencing of *MyD88* could also hinder the NFκB-c-Myc signaling cascades and phenotype transition through *FMR1-AS1* exosome incubation in the ESCC cells (Fig. 6d-f, Additional file 11: Figure S6b and Additional file 4). Moreover, we systemically investigated the effect of transmitted *FMR1-AS1* in vivo using xenograft model. We subcutaneously co-injected two types of cell mixture (ECA-109_luc & ECA-109_*FMR1-AS1* and KYSE-150_luc & KYSE-150_*FMR1-AS1*; cell number ratio = 1:5). Bioluminescent imaging revealed that the xenografts growth ability of the cell mixture containing 1/5 *FMR1-AS1* overexpressed cells were notably stronger than the control cell mixture (Fig. 6g), indicating exosomal *FMR1-AS1* could induce ESCC tumor growth in vivo. Together, these results suggest that exosomal *FMR1-AS1* induces ESCC cancer stem-like phenotypes by activating TLR7-NFκB signaling pathway, thus promoting c-Myc expression level.

## Discussion

Globally, there is a large male predominance in both esophageal squamous and adenocarcinoma. The gender difference in cancer susceptibility is one of the most consistent findings in cancer epidemiology and can give important clues for the etiology of cancers and should be examined in all genetic and non-genetic association studies. Although the incidence rate of ESCC among females is much lower than that among males, female ESCC may have specific pathogenic factors, either genetic or environmental. In this study, we identified the X-linked lncRNA, *FMR1-AS1*, which differentially expressed in female ESCC patients, as a potential female-specific genetic factor that contribute to female ESCC.

Using a large cohort of ESCC microarrays, we discovered a female specific aberrantly expressed lncRNA, *FMR1-AS1*. It is a primate-specific noncoding RNA transcript (2.4 kb) that resides upstream and shares a bidirectional promoter with *FMR1*. The CGG expansion in the 5’ UTR of *FMR1* appears not only affected on the transcription of *FMR1-AS1* and *FMR1*, but also associated with sXCI [40]. However, sXCI has been found mainly in female adults, and it is linked to the development of breast cancer [41], ovarian cancer [42] and esophageal carcinoma [24]. In the present study, we found that *FMR1-AS1* expression was more prevalent in the patients with a larger sXCI ratio, suggesting sXCI may be a risk factor associated with *FMR1-AS1* expression and that sXCI could influence the development of female ESCC. This may be another factor that contributes to the gender difference in human ESCC incidence. Our data and previous reports have demonstrated that *FMR1-AS1* greatly affected female ESCC cell proliferation and survival both in vitro and in vivo. Meanwhile, it is well known that the reprogramming of X-inactivation during acquisition of pluripotency is accompanied by the repression of *XIST*, the trigger of X-inactivation, and the upregulation of its antisense counterpart *TSIX*. Our data demonstrated that *TSIX* showed some correlation with *FMR1-AS1* and overexpressed in CD44^high^ ESCC cells and samples, indicating that sXCI within ESCC development may tend to the *FMR1-AS1* highly expressed cells to contribute female ESCC tumorigenesis and malignancy.

In the case of interconversion between CSCs and non-CSCs, exosomes may be taken granted as important signaling information transmitter by transferring stemness-related molecules to non-CSCs, which lead non-CSCs to regain stemness phenotype. Our data of ChIRP-MS and followed-up experiments indicate that *FMR1-AS1* could be selectively packaged into exosomes derived from ESCC CSCs. Cancer cells could recruit and alter phenotypic and functional attributes by uptaking exosomes. Those specific cargo molecules are needed for tumor growth, metastasis and drug resistance to enhance tumorigenicity or stemness phenotype of cancer cells. Also, our data demonstrated that *FMR1-AS1* could interact with the Toll-like Receptor 7, TLR7. TLR7 was identified as one of the PRRs-sensing exosomes that contribute to the stimulation of downstream pathways in breast cancer cells [31]. Intriguingly, TLR7 documented as a member of endosomal TLRs. And gender difference in TLR7 response has been reported previously in human immune diseases [43, 44]. Our study also showed that sXCI may contribute to the *FMR1-AS1*-dependent TLR7 responses in female ESCC cells. Further, recent studies have shown that TLRs are expressed in a variety of tumor cells through the MyD88 pathway to ultimately activate NFκB, stimulating stem cell associated factors and pathways, especially c-Myc within Wnt and Notch signaling [45–47]. c-Myc is also among the several stemness factors that could direct promote *TSIX* expression [48]. Previous research has examined the contribution of exosomes to cancer population equilibrium and tumor heterogeneity, in which transitions between clonogenic states could be modulated by exosome-mediated Wnt signaling [49]. Our results showed that *FMR1-AS1* notably overexpressed in the CD44^+^ cancer stem-like cells within female ESCC patients. Transfer of *FMR1-AS1* or FMR1-AS1 overexpressed ESCC cell derived exosomes to wild-type ESCC cell resulted in aggressive phenotypes, including elevated proliferation, anti-apoptosis, migration and invasion ability. And these responses were abolished in MyD88 or TLR7 deficient cells suggesting that exosomes containing *FMR1-AS1* derived from ESCC CSCs could activate TLR7-MyD88-NFκB-c-Myc signaling pathways in non-CSCs involved with stemness phenotypes while the release and uptake of exosome could be controlled. In general, several reports and our findings together indicate that exosomes derived from aggressive cancer cells, especially the CSCs could transport oncogenic factors, including lncRNAs, to recipient cells within tumor microenvironment to induce tumor aggression and progression.

**Fig. 7:**
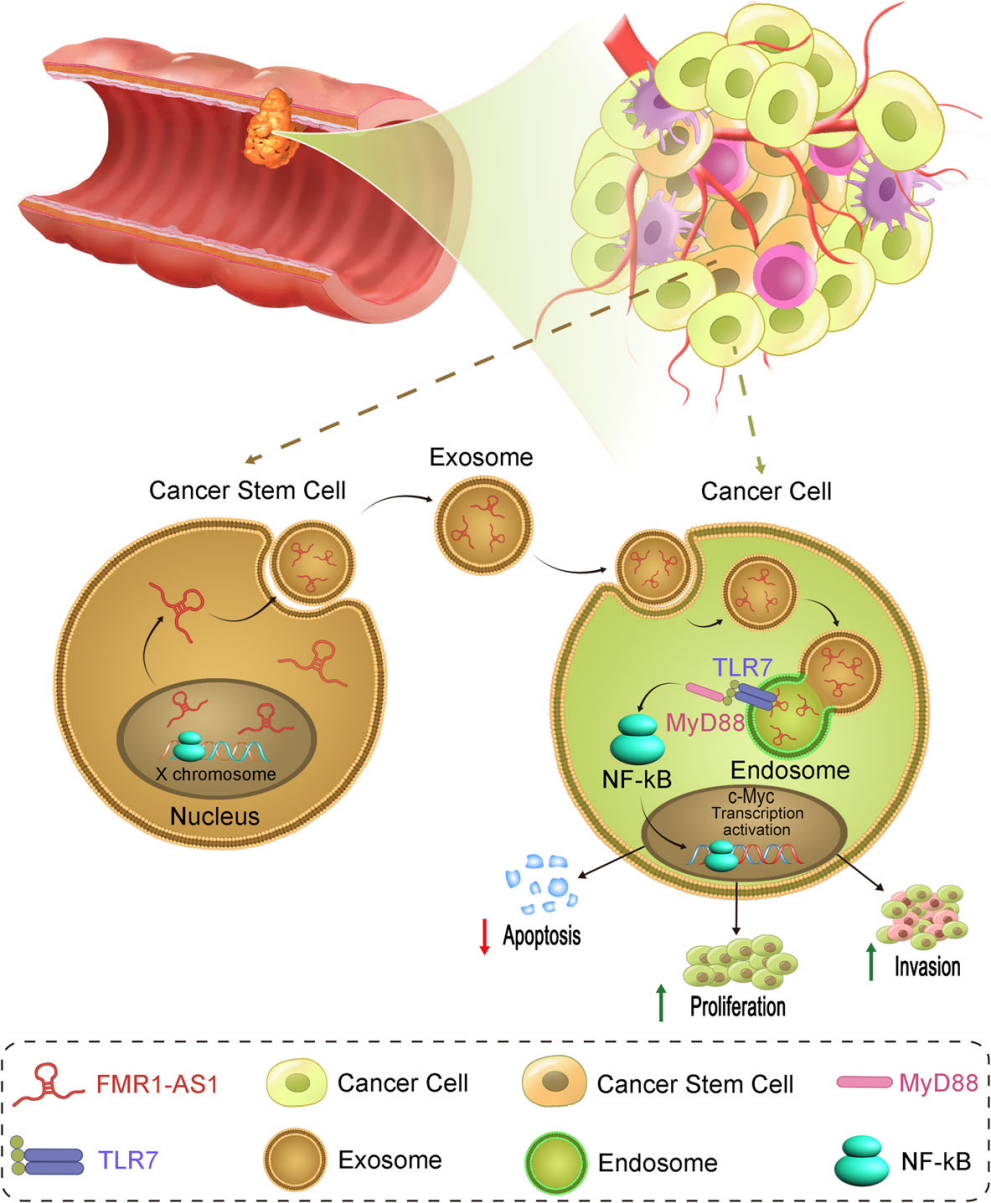
A schematic diagram of *FMR1-AS1* mediated TLR7-NFκB signaling activation between cancer stem-like cells and cancer cells of female ESCC

In sum, the sex-dependent *FMR1-AS1* within exosomes secreted by ESCC cancer stem-like cells can bind to endosomal TLR7 and activate TLR7-NFκB signaling, thus promoting the expression of downstream CSC-linked gene, c-Myc, in the recipient non-CSCs (Fig. 7). This mechanism of *FMR1-AS1* is implicated in tumor intrinsic communication and contribute to ESCC development.

## Conclusions

Overall, our study could, to some extent, broaden the understanding of the complex processes of tumor cell heterogeneity and highlight exosomes as a key player in this process, in which case a comprehensive therapeutic strategy targeting exosomes to eradicate CSCs for cancer therapeutics would be more effective.

## Additional files

Additional file 1: **Table S1.** Distributions of characteristics among female ESCC patients in Chinese populations used for study. (DOCX 18 kb)

Additional file 2: Materials and Methods. (DOCX 34 kb)

Additional file 3: **Figure S1.** Female specific X-associated lncRNA screening and *FMR1-AS1* expression patterns in female ESCC samples and cells. (TIF 339 kb)

Additional file 4: Supplementary figure legends. (DOCX 17 kb)

Additional file 5: **Figure S2.** Biological characterization of *FMR1-AS1*. (TIF 7610 kb)

Additional file 6: **Figure S3.** Effects of ectopic *FMR1-AS1* expression on female ESCC cells. (TIF 5856 kb)

Additional file 7: **Table S2.** Information of primers and probes. (XLSX 15 kb)

Additional file 8: **Table S3.** Results of mass spectrum. (XLSX 23 kb)

Additional file 9: **Figure S4.** *FMR1-AS1* could be packaged into exosomes and activates TLR7- NFκB-c-Myc signaling. (TIF 1679 kb)

Additional file 10: **Figure S5.** Intercellular transfer of *FMR1-AS1* by exosomes disseminates ESCC stem-like phenotypes. (TIF 1036 kb)

Additional file 11: **Figure S6.** TLR7-NFκB signaling pathway activation is responsible for *FMR1-AS1*-mediated cancer stem cell transition. (TIF 4009 kb)

## Abbreviations

CICs: Cancer-initiating cells
CR: Corrected ratio
CSCs: Cancer stem cells
ESCC: Esophageal squamous cell carcinoma
LncRNA: Long non-coding RNA
PRRs: Pattern recognition receptors
TFBS: Transcription factor binding sites
TLR7: Toll-like receptor 7
