# Supplementary material for: Exosomal FMR1-AS1 facilitates maintaining cancer stem-like cell dynamic equilibrium via TLR7/NFκB/c-Myc signaling in female esophageal carcinoma
Source: Mol Cancer. 2019 Feb 8;18:22. doi: 10.1186/s12943-019-0949-7 (PMC6367809; doi:10.1186/s12943-019-0949-7)
Supplement: Supplementary file 1 — Table S1. Distributions of characteristics among female ESCC patients in Chinese populations used for study. (DOCX 18 kb) [file 12943_2019_949_MOESM1_ESM.docx]

**Table s1. Distributions of characteristics among female ESCC patients in Chinese populations used for study.**

| **Characteristics** | | **Suzhou population** | |  | **Guangzhou population** | |  | **Overall** | |
| --- | --- | --- | --- | --- | --- | --- | --- | --- | --- |
|  |  | **N** | **(%)** |  | **N** | **(%)** |  | **N** | **(%)** |
| **Sex** | |  |  |  |  |  |  |  |  |
|  | Female | 206 | (100) |  | 188 | (100) |  | 394 | (100) |
| **Age(years)** | |  |  |  |  |  |  |  |  |
|  | ≤40 | 19 | (9.2) |  | 16 | (8.5) |  | 35 | (8.9) |
|  | 40-60 | 129 | (62.6) |  | 103 | (54.8) |  | 232 | (58.9) |
|  | ≥60 | 58 | (28.2) |  | 69 | (36.7) |  | 127 | (32.2) |
| **Body mass index** | |  |  |  |  |  |  |  |  |
|  | ≤20 | 39 | (18.9) |  | 37 | (19.7) |  | 76 | (19.3) |
|  | 20-28 | 143 | (69.4) |  | 126 | (67.0) |  | 269 | (68.3) |
|  | ≥28 | 24 | (11.7) |  | 25 | (13.3) |  | 49 | (12.4) |
| **Family history** | |  |  |  |  |  |  |  |  |
|  | Positive | 28 | (13.6) |  | 17 | (9.0) |  | 45 | (11.4) |
|  | Negative | 178 | (86.4) |  | 171 | (91.0) |  | 349 | (88.6) |
| **Smoking** | |  |  |  |  |  |  |  |  |
|  | Positive | 56 | (27.2) |  | 57 | (30.3) |  | 113 | (28.7) |
|  | Negative | 150 | (72.8) |  | 131 | (69.7) |  | 281 | (71.3) |
| **Drinking** | |  |  |  |  |  |  |  |  |
|  | Positive | 69 | (33.5) |  | 73 | (38.8) |  | 142 | (36.0) |
|  | Negative | 137 | (66.5) |  | 115 | (61.2) |  | 252 | (64.0) |
| **Pathological type** | |  |  |  |  |  |  |  |  |
|  | Highly differentiated | 24 | (11.6) |  | 21 | (11.2) |  | 45 | (11.4) |
|  | Moderately differentiated | 97 | (47.1) |  | 103 | (54.8) |  | 200 | (50.8) |
|  | Low differentiated | 85 | (41.3) |  | 64 | (34.0) |  | 149 | (37.8) |
| **Stage** | |  |  |  |  |  |  |  |  |
|  | I | 22 | (10.7) |  | 19 | (10.1) |  | 41 | (10.4) |
|  | II | 99 | (48.0) |  | 84 | (44.7) |  | 183 | (46.5) |
|  | III | 85 | (41.3) |  | 85 | (45.2) |  | 170 | (43.1) |
