# Supplementary material for: Exosomal FMR1-AS1 facilitates maintaining cancer stem-like cell dynamic equilibrium via TLR7/NFκB/c-Myc signaling in female esophageal carcinoma
Source: Mol Cancer. 2019 Feb 8;18:22. doi: 10.1186/s12943-019-0949-7 (PMC6367809; doi:10.1186/s12943-019-0949-7)
Supplement: Supplementary file 2 — Materials and Methods. (DOCX 34 kb) [file 12943_2019_949_MOESM2_ESM.docx]

**Supplementary Material and Methods**

**Study approval**

Ethical consent was given by Soochow University Committee for Ethical Review of Research Involving Human Subjects. The use of human ESCC cancer tissue specimens was evaluated and approved by the Ethical Committee of the Affiliate Hospitals of Soochow University and the Cancer Hospitals affiliated with Guangzhou Medical University, and written informed consent was obtained from all participants or their appropriate surrogates. All animal studies were conducted with the approval of Soochow University Institutional Animal Care and Use Committee and were performed in accordance with established guidelines.

**Cell lines**

The human ESCC cell lines ECA-109, KYSE-150 and the embryonic kidney cell line 293T were purchased from Cell Bank of Chinese Academy of Sciences. ECA-109, KYSE-150 and 293T cells were maintained in in RPMI-1640 medium supplemented with 10% fetal calf serum (Invitrogen). All cell lines were grown in an atmosphere of 5% CO2 and 99% relative humidity at 37°C. Cell lines were passaged for fewer than 6 months and were authenticated by short tandem repeat analysis. No mycoplasma infection was found for all cell lines.

TNF-α (Peprotech, Rocky Hill, NJ) was used to treat cells in the concentration of 10ng/ml for 24h. To inhibit NFκB nuclear translocation, 10μM sc-3060 (Santa Cruz, Santa Cruz, CA) or 5μM JSH-23 (481408, Millipore, Billerica, MA) were added to culture media 30min prior to the specified treatments.

**RNA Extraction and Quantitative Real-time PCR Analysis**

Total RNA from the endometrial tissue specimens and from cells was extracted with TRIzol reagent (Invitrogen, Carlsbad, CA, USA). First-strand cDNA was synthesized with the Superscript II-reverse transcriptase kit (Invitrogen, Carlsbad, CA, USA). RNA levels were determined by quantitative real-time PCR (qRT-PCR) in triplicate on a Roche Light Cycler 480 using the SYBR Green method. All qRT-PCR primers are listed in **Additional file 3: Table s2**. The RNA level was normalized against β-ACTIN or GAPDH RNA using the comparative Ct method.

**Cell fractionation RNA and fluorescence in situ hybridization (FISH)**

To determine the cellular localization of *FMR1-AS1*, cell fractionation was performed as previously described [[1](#_ENREF_1)]. Briefly, cells were resuspended in buffer 1 (50 mM Tris-HCl pH 8, 140 mM NaCl, 1.5 mM MgCl2, 0.5% NP-40) supplemented with SUPERase•In (Ambion) for 10 min on ice. After a centrifugation at 300g for 2 min, the supernatant was collected as the cytoplasmic fraction. The pellet was resuspended in buffer 2 (50 mM Tris-HCl pH 8, 500 mM NaCl, 1.5 mM MgCl2, 0.5% NP-40) supplemented with RNase inhibitors for 10 min on ice. Chromatin was pelleted at maximum speed for 3 min. The supernatant represents the nuclear fraction. RNA was extracted following the standard protocol. RNA FISH experiments were performed with FISH Detection Kit (Ribo, Guangzhou, China) in ESCC cell lines according to the protocol.

**Northern blotting**

Northern-blot was performed using a Roche DIG Northern Starter Kit (Roche, Germany) following the manufacturer’s instructions. A total of 10 μg of the indicated RNA was subjected to formaldehyde gel electrophoresis and transferred to a Biodyne Nylon membrane (Pall, NY). The PCR primers were listed in **Supplementary Table 3**.

**Analysis of skewed X chromosome inactivation (SXCI)**

DNA was digested by mixing 10 μl (1 μg) of sample DNA with 0.5μL of HpaII (10 U/μL; Promega, Madison, WI, USA), 2 μl of 10 mol/L reaction buffer, 0.2μL of 10 g/L bovine serum albumin and 7.3 μl of deionized water. The mixture was then incubated at 37°C for 4 h and the reaction was terminated by incubation at room temperature for 30 min as suggested by the manufacturer. Nested PCR was conducted as described previously [[2](#_ENREF_2)]. A negative, water-blank control was always included in each batch of PCR. If the negative control was shown to be positive, the reaction was repeated for the whole batch. The reaction fidelity of HpaII digestion was guaranteed by parallel negative controls with the enzyme omitted from the reaction mixture. In addition, the whole assay was carried out twice in independent series.

Amplification efficacy was demonstrated through electrophoresis on 2% agarose gels. The amplification products with 4 μL for each were mixed with the same volume of loading buffer (1g/L xylene cyanole, 1g/L bromophenol blue, in formamide), loaded onto the 10% polyacrylamide gel containing 8 mol/L urea, resolved through electrophoresis with the Mini-VE system (Amersham Biosciences Corp., San Francisco, CA) at a voltage of 80 v for 8 h, and then visualized after silver staining as described previously[[3](#_ENREF_3)]. For the samples whose allelic differences at the CAG STR were small (one or two repeats), a longer gel (26-cm long and 0.75-mm thick) was used for the resolution with the SE660 system (Amersham). The results were recorded, and the intensities of the products from both alleles were analyzed by using an image-analyzing system (LabWorks 3.0, UVP, Cambridge, UK).

In order to avoid the interference of possible preferential amplification of one of the alleles, we used the corrected ratio (CR) to evaluate the X-chromosome inactivation pattern by comparing the allelic difference of a sample before and after HpaII digestion. CR was derived by dividing the ratio of the upper-band intensity to the lower-band intensity of the sample after digestion by that of the same sample before digestion. If CR was <1, the reciprocal value was considered. In the present study, CR ≥ 3, which indicated the expression of the same allele in above 75% of the cells examined, was used to define SXCI. In addition, we also used CR ≥ 10 as a more stringent criterion for defining SXCI.

**Comprehensive identification of RNA-binding proteins by mass spectrometry (ChIRP-MS)**

10-20 15cm dishes of cells were used per ChIRP-MS experiment (100million - 500million cells depending on the cell type). Cell harvesting, lysis, disruption, and ChIRP were essentially performed as previously described [[4](#_ENREF_4)], with the following modifications: 1) Cells are crosslinked in 3% formaldehyde for 30min, followed by 0.125M glycine quenching for 5min; 2) hybridization can be started late in the day and left running overnight to reduce hands-on time; 3) For mass spec experiments, lysates were pre-cleared by incubating with 30μL washed beads per ml of lysate at 37°C for 30min with shaking. Prior to hybridization beads were removed twice from lysate using a magnetic stand; 4) for RNase control, lysates are pooled first and aliquoted into two equal amounts. 1/1000 volume of 10mg/ml Rnase A (Sigma) is added to the RNase control sample and both control and non-treated samples are incubated at 37°C for 30min with mixing prior to hybridization steps. This can be done concurrently with pre-clearing. RNA extraction can be performed from a small aliquot of post-ChIRP beads as described (Chu et al., 2012). For protein elution, beads were collected on magnetic stand, resuspended in biotin elution buffer (12.5mM biotin (Invitrogen), 7.5mM HEPES pH 7.5, 75mM NaCl, 1.5mM EDTA, 0.15% SDS, 0.075% sarkosyl, and 0.02% Na-Deoxycholate), mixed at r.t. for 20min, and at 65°C for 10min. Eluent was transferred to a fresh tube and beads were eluted again. The two eluents were pooled, and residual beads were removed again using the magnetic stand. ¼ total volume TCA was added to the clean eluent, and after thorough mixing proteins were precipitated at 4°C overnight. Next day, proteins were pelleted at 16000rcf at 4°C for 30min. Supernatant was carefully removed from the belly side of tubes and protein pellets on the spine of tubes (sometimes invisible at this step) were washed once with cold acetone and pelleted again at 16000rcf at 4°C for 5min and acetone was removed. Pellets (much more visible now) were briefly centrifuged again and after removal of residual acetone, left to air-dry for 1 min on bench-top. Proteins are then immediately solubilized in desired volumes of 1×laemmli sample buffer (Invitrogen), boiled at 95°C for 30min with occasional mixing for reverse-crosslinking. Final protein samples were size-separated in bis-tris SDS-PAGE gels (Invitrogen) for western blots or MS. The following antibodies were used in the followed western-blotting: anti-TLR7 (ab45371, Abcam), anti-hnRNPK (ab39975, Abcam), anti-PRDX1 (ab41906, Abcam), anti-PRDX2 (ab109367, Abcam), anti-ECM1 (ab126629, Abcam) and anti-β-actin (ab8227, Abcam).

**RNA pull-down and RNA immunoprecipitation**

RNA pull-down assays were performed as previously described [[5](#_ENREF_5)]. Briefly, biotinylated *FMR1-AS1* or its antisense RNA was incubated with cellular protein extracts, which were then treated with streptavidin beads. Recovered proteins that were associated with *FMR1-AS1* were resolved by gel electrophoresis. *FMR1-AS1* and its fragments were transcribed *in vitro* with primers that contained a T7 promoter sequence. The following antibodies were used: anti-TLR7 (ab45371, Abcam) and anti-β-actin (ab8227, Abcam). We performed RNA immunoprecipitation (RIP) experiments using a Magna RIP Kit (Millipore, Bedford, MA) following the manufacturer’s instructions. The following antibodies were used for RIP: anti-TLR7 (ab45371, Abcam), anti-TLR2 (ab9100, Abcam), anti-TLR3 (ab13915, Abcam), anti-TLR4 (ab13556, Abcam), anti-TLR8 (ab180610, Abcam) and IgG control (sc-2027, Santa Cruz Biotechnology).

**Plasmids, lentiviral production and shRNAs**

The full-length human *FMR1-AS1* cDNA was synthesized and cloned into the lentiviral expression vectors by Applied Biological Materials Inc (Jiangsu, China). The lentiviral shRNA of human *FMR1-AS1*, *TLR7* and *MyD88* was also provided by Applied Biological Materials Inc. (Jiangsu, China). The resultant construct was verified by sequencing.

**Construction of reporter plasmids, transient transfections and luciferase assays**

The fragment was synthesized and then inserted into the pGL3-basic vector (Promega, Madison, WI) (termed the pGL3-promoter). All constructs were sequenced to verify the allele, orientation and integrity of each insert.

**Chromatin immunoprecipitation and EMSA**

ChIP assays were performed with an EZ-ChIP kit (Millipore, Bedford, MA) following the manufacturer’s instructions. Chromatin from ESCC cells was immunoprecipitated with antibodies against p65 (ab16502, Abcam), p50 (ab32360, Abcam), RNAP Ⅱ(ab817, Abcam) and IgG control (sc-2027, Santa Cruz Biotechnology).

EMSA Assay was performed using LightShift Chemiluminescent EMSA Kit (20148, Pierce, Rockforld, IL) according to manufacturer’s instructions.

**Antibodies and Western blotting**

Protein lysates from specific ESCC cells were subjected to Western blot analysis by using anti-β-catenin (ab32572, Abcam), anti-c-myc (ab32072, Abcam), anti-c-jun (ab31419, Abcam), anti-activated Notch1 (NICD antibody) (ab8925, Abcam), Anti-HES1 (ab71559, Abcam), and Anti-p21(ab7960, Abcam) according to standard protocols as previously described. Protein expression levels were normalized to that of β-actin by calculating the relative expression levels. Anti-CD44 (ab157107, Abcam) was used in the tissue immunostaining and cell flow sorting of ESCC samples.

**Flow cytometry analysis of apoptosis, xenograft TUNEL and KI67 staining**

For apoptosis analysis, Annexin V-FITC/PI staining was performed by using flow cytometry according to the manufacturer’s guidelines (Roche, German). Xenograft tumors were collected on Day 16. The level of proliferation and cell survival in the xenograft tissues were detected by Ki67 (ab15580, Abcam) immunostaining and TUNEL staining according to the manufacturer’s instructions (Roche, German).

**Analysis of cell proliferation, cell cycle, wound healing and colony formation ability**

For cell viability, cells were seeded in 96-well flat-bottomed plates and then measured with a CCK-8 assay (Dojindo, Japan). For cell cycle, cells fixed in 70% ethanol and labeled with propidium iodide (PI, Sigma), then were analyzed by flow cytometry (Beckman Coulter). For wound-healing assay, cells were treated with mitomycin C and grown to confluence as a monolayer, then wounded with a 10μl pipette tip in non-serum medium. For colony formation assay, cells were seeded in 65mm culture dishes and were allowed to grow until visible colonies formed (2 weeks). All experiments were repeated at least three times.

**Transwell migration and Matrigel invasion assays**

The ability of the cells to migrate and invade was assessed using Corning Transwell insert chambers with pores 8 mm in size (Corning) and a BD BioCoat Matrigel Invasion Chamber (Becton Dickinson Biosciences), respectively. Approximately 1×10^4^ (migration assay) or 2×10^5^ (invasion assay) transfected cells in 200 μl of serum-free RPMI 1640 medium were seeded in the upper well; the chambers were then incubated with RPMI 1640 medium plus 20% fetal bovine serum for 48 h at 37°C to allow the cells to migrate to the lower well. The cells that had migrated or invaded through the membrane were fixed in methanol, stained with crystal violet (Invitrogen), imaged and counted.

**Exosome Isolation and Application**

For exosome purification, culture medium was pre-cleared by filtration through a 0.22μm PVDF filter (Millipore, USA). Exosomes were collected through standard centrifugation steps as previously described. Exosomes were examined by Electron Microscopy using negative staining and quantified by NanoSight NS300 instrument (Malvern Instruments Ltd. UK) equipped with NTA 3.0 analytical software (Malvern Instruments Ltd. UK). The antibodies of CD63 (ab59479, Abcam) and CD81 (ab79559, Abcam) were used as surface markers in the FACS detection of exosome.

For exosomal RNA and protein extraction, exosomes were pre-treated with RNase or Proteinase K respectively. Equal number of exosomes were used for RNA extraction and normalized against exogenous λ polyA (Takara, China) for qPCR; equal number of exosomes used for protein extraction were suspended in SDS lysis buffer and quantified by bicinchoninic acid assays (Pierce, USA).

For in vitro exosome treatment, 1μg exosomes (equivalent to those collected from ~5×10^6^ producer cells) were added to 2×10^5^ recipient cells. For *in vivo* exosome treatment, exosomes were injected intratumorally every two days (5 μg exosomes per injection).

**Gene set enrichment analysis (GSEA)**

GSEA was performed by the JAVA program (http://www.broadinstitute.org/gsea) using MSigD. Canonical pathways gene set collection (1320 gene sets available). Gene sets with a false discovery rate (FDR) value <0.05 after performing 1,000 permutations were considered to be significantly enriched [[6](#_ENREF_6)]. Cytoscape and Enrichment Map were used for visualization of the GSEA results. GSEA is a computational method that determines whether a priori defined set of genes shows statistically significant concordant differences between two biological states. GSEA is a powerful analytical method for interpreting gene expression data and GSEA focuses on genes that share common biological function, chromosomal location, or regulation. GSEA was performed by the GSEA desktop application with default parameters.

**Accession numbers**

The accession number for the microarray data analyzed in this paper is Gene Expression Omnibus database GEO: GSE53625 and GSE70817. The ribosome profiling data were obtained from the GSE61742.

**References**

1. Ranzani V, Rossetti G, Panzeri I, Arrigoni A, Bonnal RJ, Curti S, Gruarin P, Provasi E, Sugliano E, Marconi M, et al: **The long intergenic noncoding RNA landscape of human lymphocytes highlights the regulation of T cell differentiation by linc-MAF-4.** *Nat Immunol* 2015, **16:**318-325.

2. Li G, Su Q, Liu GQ, Gong L, Zhang W, Zhu SJ, Zhang HL, Feng YM: **Skewed X chromosome inactivation of blood cells is associated with early development of lung cancer in females.** *Oncol Rep* 2006, **16:**859-864.

3. Lucas DR, Shroyer KR, McCarthy PJ, Markham NE, Fujita M, Enomoto TE: **Desmoid tumor is a clonal cellular proliferation: PCR amplification of HUMARA for analysis of patterns of X-chromosome inactivation.** *Am J Surg Pathol* 1997, **21:**306-311.

4. Chu C, Quinn J, Chang HY: **Chromatin isolation by RNA purification (ChIRP).** *J Vis Exp* 2012.

5. Rinn JL, Kertesz M, Wang JK, Squazzo SL, Xu X, Brugmann SA, Goodnough LH, Helms JA, Farnham PJ, Segal E, Chang HY: **Functional demarcation of active and silent chromatin domains in human HOX loci by noncoding RNAs.** *Cell* 2007, **129:**1311-1323.

6. Subramanian A, Tamayo P, Mootha VK, Mukherjee S, Ebert BL, Gillette MA, Paulovich A, Pomeroy SL, Golub TR, Lander ES, Mesirov JP: **Gene set enrichment analysis: a knowledge-based approach for interpreting genome-wide expression profiles.** *Proc Natl Acad Sci U S A* 2005, **102:**15545-15550.
