# Supplementary figures and images for: Exosomal FMR1-AS1 facilitates maintaining cancer stem-like cell dynamic equilibrium via TLR7/NFκB/c-Myc signaling in female esophageal carcinoma
Source: Mol Cancer. 2019 Feb 8;18:22. doi: 10.1186/s12943-019-0949-7 (PMC6367809; doi:10.1186/s12943-019-0949-7)

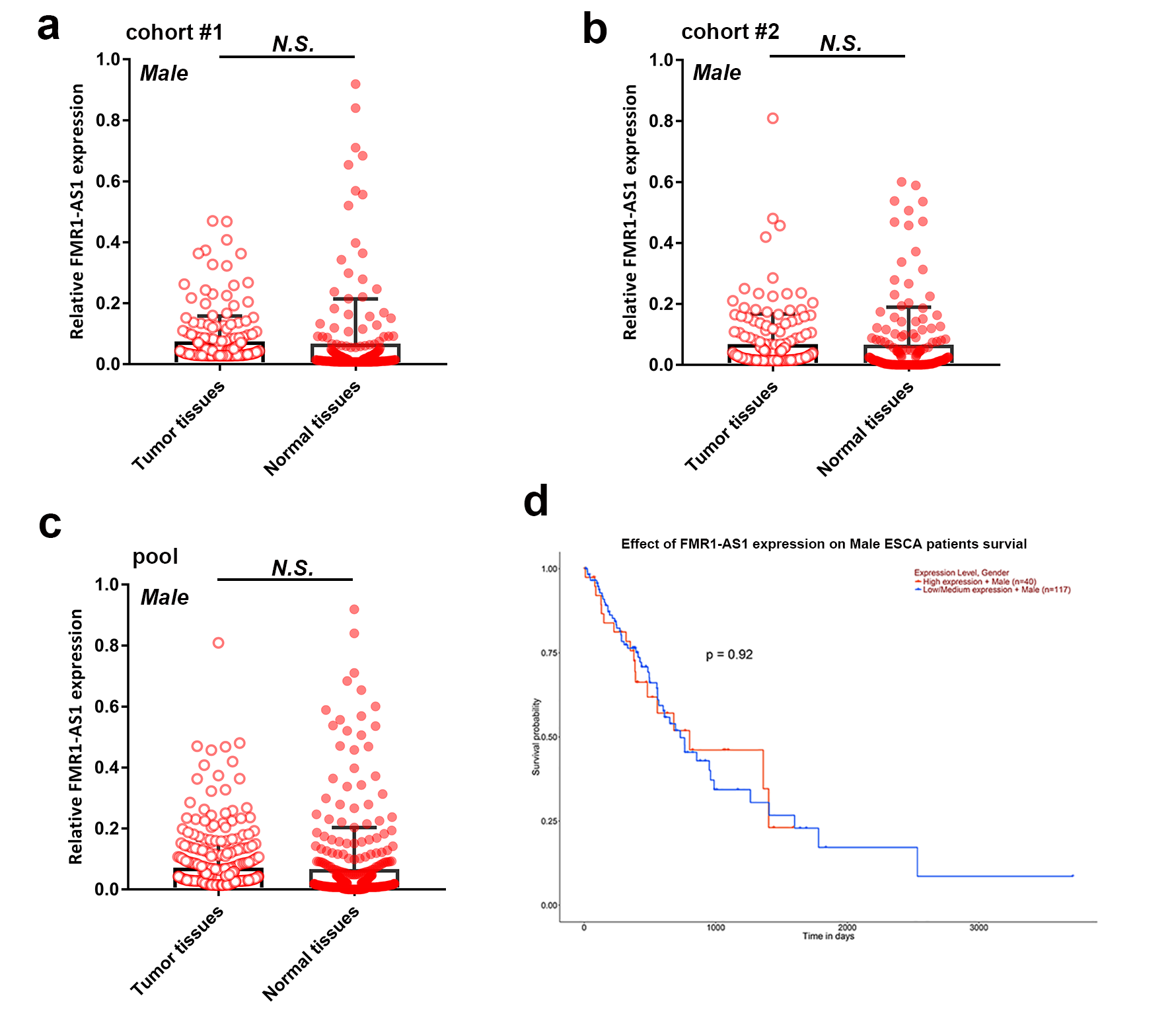

Supplement: Supplementary file 3 — Figure S1. Female specific X-associated lncRNA screening and FMR1-AS1 expression patterns in female ESCC samples and cells. (TIF 339 kb) [file 12943_2019_949_MOESM3_ESM.tif]

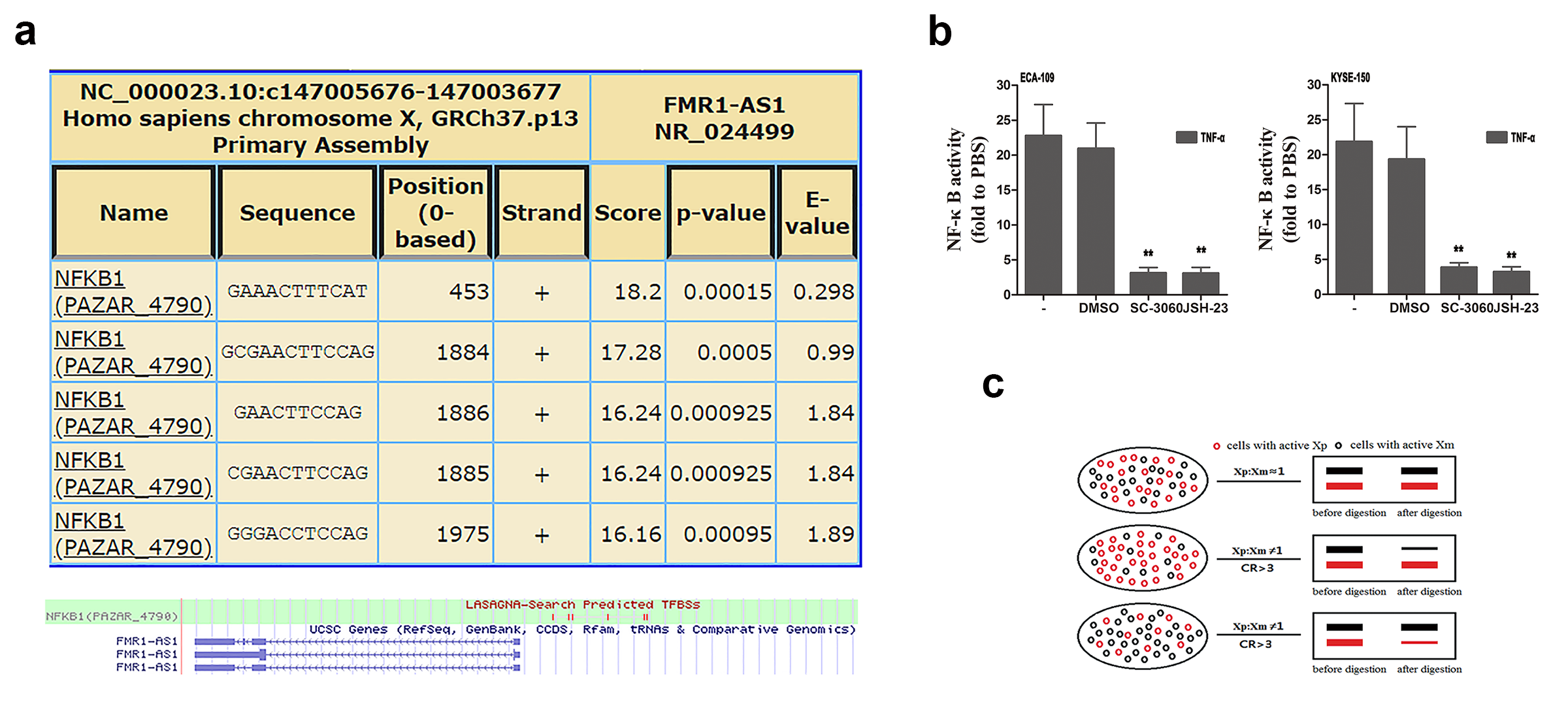

Supplement: Supplementary file 5 — Figure S2. Biological characterization of FMR1-AS1. (TIF 7610 kb) [file 12943_2019_949_MOESM5_ESM.tif]

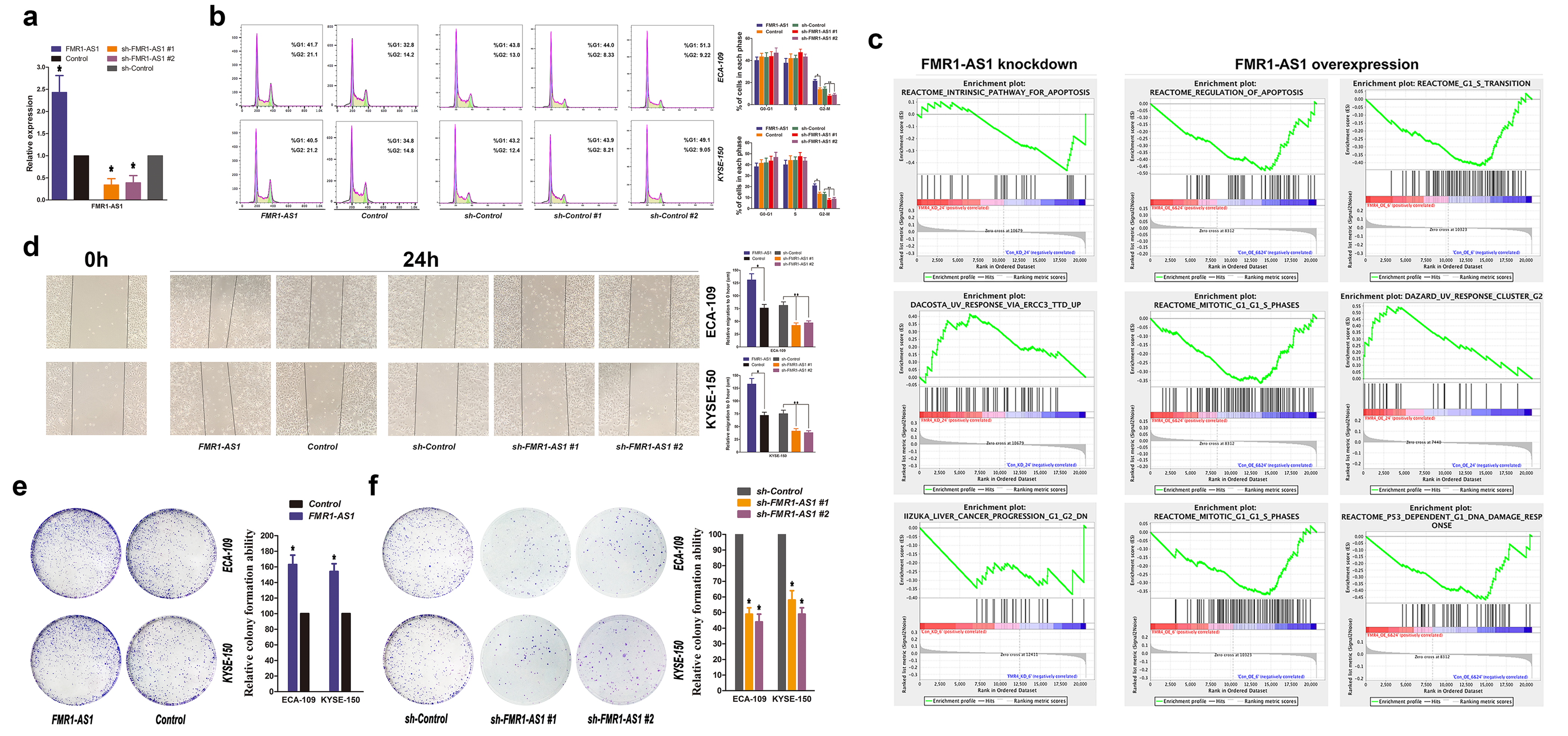

Supplement: Supplementary file 6 — Figure S3. Effects of ectopic FMR1-AS1 expression on female ESCC cells. (TIF 5856 kb) [file 12943_2019_949_MOESM6_ESM.tif]

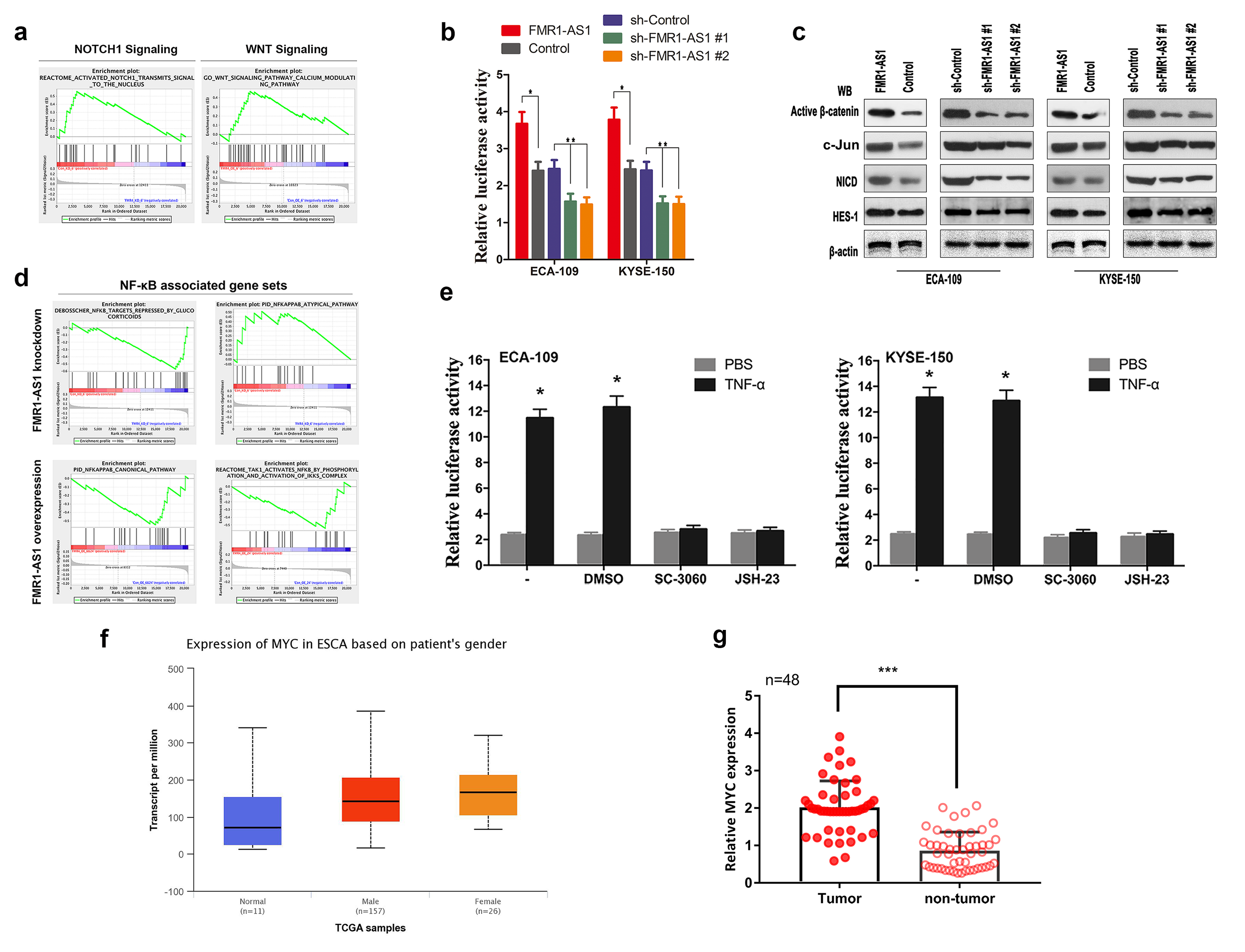

Supplement: Supplementary file 9 — Figure S4. FMR1-AS1 could be packaged into exosomes and activates TLR7- NFκB-c-Myc signaling. (TIF 1679 kb) [file 12943_2019_949_MOESM9_ESM.tif]

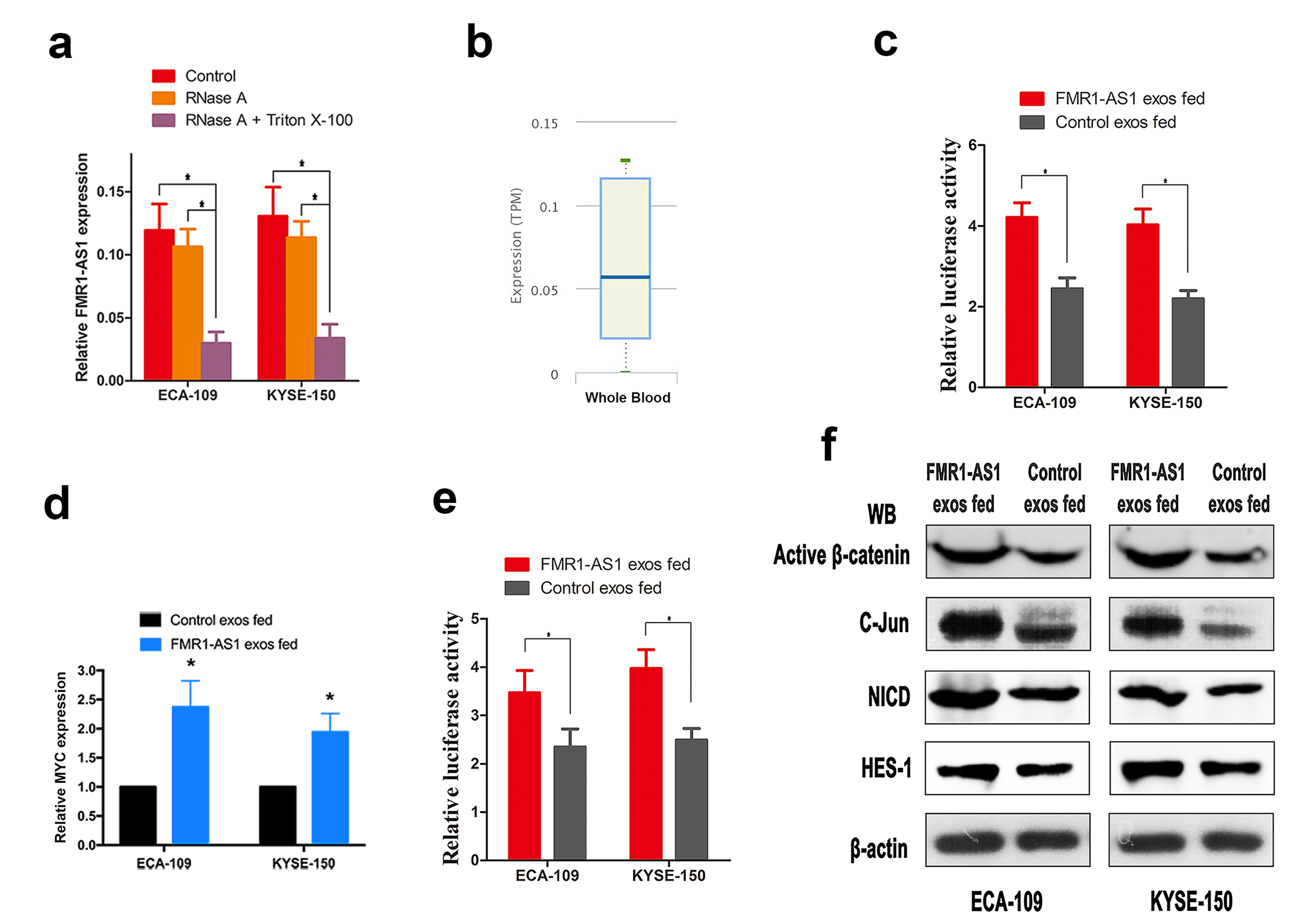

Supplement: Supplementary file 10 — Figure S5. Intercellular transfer of FMR1-AS1 by exosomes disseminates ESCC stem-like phenotypes. (TIF 1036 kb) [file 12943_2019_949_MOESM10_ESM.tif]

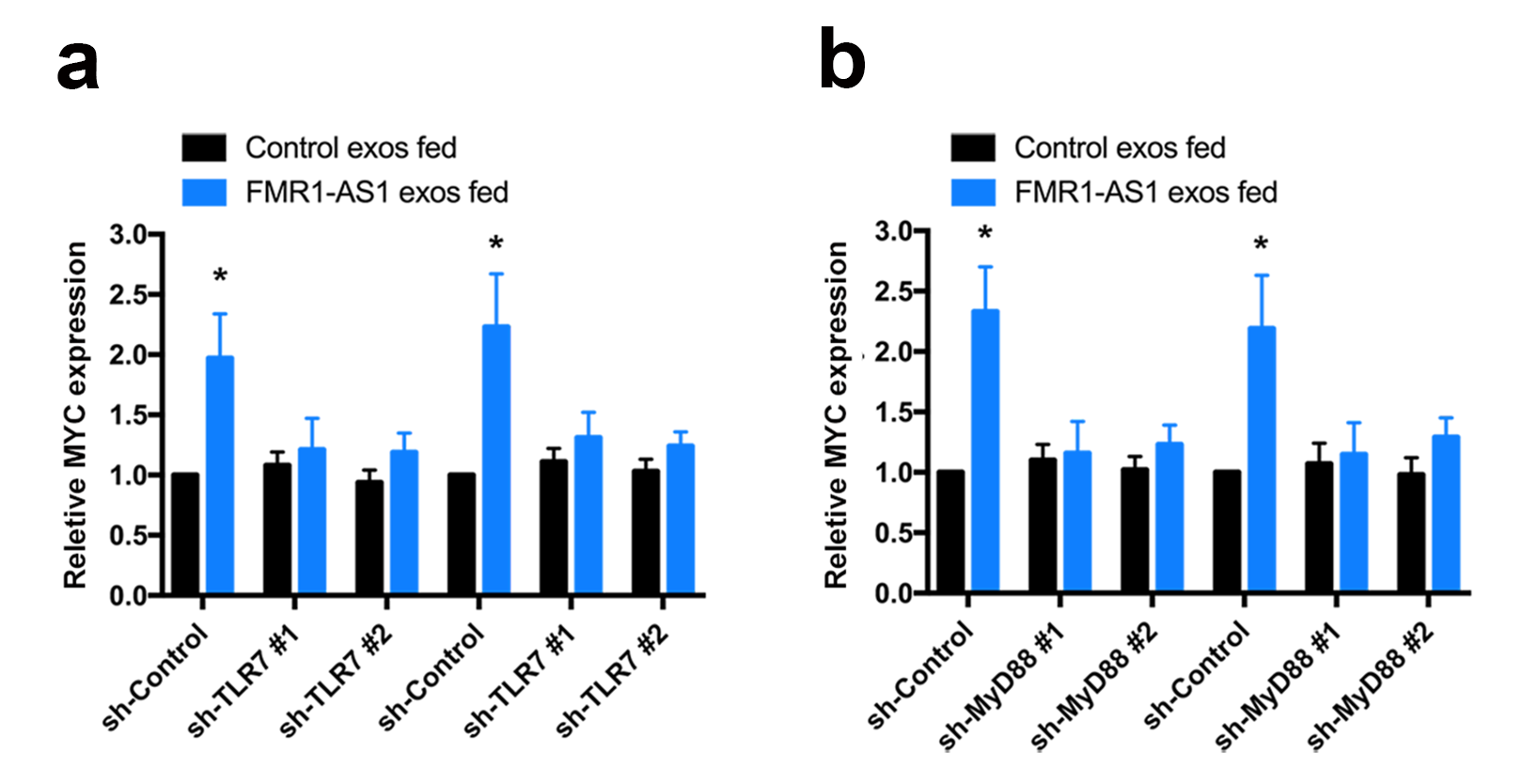

Supplement: Supplementary file 11 — Figure S6. TLR7-NFκB signaling pathway activation is responsible for FMR1-AS1-mediated cancer stem cell transition. (TIF 4009 kb) [file 12943_2019_949_MOESM11_ESM.tif]
