# Supplementary material for: Exosomal FMR1-AS1 facilitates maintaining cancer stem-like cell dynamic equilibrium via TLR7/NFκB/c-Myc signaling in female esophageal carcinoma
Source: Mol Cancer. 2019 Feb 8;18:22. doi: 10.1186/s12943-019-0949-7 (PMC6367809; doi:10.1186/s12943-019-0949-7)
Supplement: Supplementary file 4 — Supplementary figure legends. (DOCX 17 kb) [file 12943_2019_949_MOESM4_ESM.docx]

**SUPPLEMENTARY FIGURE LEGENDS**

**Figure s1.**

**a, b, c** *FMR1-AS1* expression in male ESCC and matched non-tumor tissues from Suzhou (n=192) and Guangzhou (n=168).

**d** Kaplan-Meier plots for survival rate of male ESCC patients in groups of *FMR1-AS1* high or low expression levels from TCGA data.

**Figure s2.**

**a** NFκB binding motifs within *FMR1-AS1* promoter region predicted by LASAGNA-Search 2.0 and visualized in UCSC genome browser.

**b** NFκB activity of TNF-α-treated ECA-109 and KYSE-150 cells, examined by luciferase

reporter assay (mean±SD, n=4, ***p*<0.01, versus DMSO), with or without NFκB inhibition by sc-3060 or JSH-23.

**c** Schematic diagram of SXCI analysis.

**Figure s3.**

**a** *FMR1-AS1* expression levels detected by qPCR in ECA-109 and KYSE-150 cells transfected with *FMR1-AS1*, Control, sh-Control, sh-*FMR1-AS1* #1 and sh-*FMR1-AS1* #2 lentiviruses. Data shown are the mean±SD of 3 independent experiments, normalized to GAPDH (**p*<0.05).

**b** Cell-cycle analysis of ECA-109 and KYSE-150 cells transfected with *FMR1-AS1*, Control, sh-Control, sh-*FMR1-AS1* #1 and sh-*FMR1-AS1* #2 lentiviruses. Results are represented as mean ± SD based on 3 independent experiments (**p*<0.05).

**c** GSEA of apoptosis associated gene signatures in *FMR1-AS1* upregulated and downregulated cells, compared to respective control cells. NES, normalized enrichment score.

**d** Wound-healing assay using ECA-109 and KYSE-150 cells transfected with *FMR1-AS1*, Control, sh-Control, sh-*FMR1-AS1* #1 and sh-*FMR1-AS1* #2 lentiviruses. Right panel is the quantification of the relative migration distance. Asterisk indicates a significant change (n=3, *p*<0.05).

**e** Representative colony formation assay in ECA-109 and KYSE-150 cells transfected with *FMR1-AS1* and Control lentiviruses. Right panel is the quantitative analysis of colony formation. Colony numbers of control cells were set to 100%. Values are expressed as mean ± SD from 3 experiments (**p*<0.05).

**f** Representative colony formation assay in ECA-109 and KYSE-150 cells transfected with sh-Control, sh-*FMR1-AS1* #1 and sh-*FMR1-AS1* #2 lentiviruses. Right panel is the quantitative analysis of colony formation. Colony numbers of control cells were set to 100%. Values are expressed as mean±SD from 3 experiments (**p*<0.05).

**Figure s4.**

**a** GSEA of cancer stem cell associated gene signatures in *FMR1-AS1* upregulated and downregulated cells, compared to respective control cells.

**b** TOP-FLASH reporter assay in ESCC cells stably transfected with *FMR1-AS1*, Control, sh-Control, sh-*FMR1-AS1* #1 and sh-*FMR1-AS1* #2 lentiviruses.

**c** Western blotting showing active β-catenin, c-Jun, NICD and HES-1levels in ESCC cells stably transfected with *FMR1-AS1*, Control, sh-Control, sh-*FMR1-AS1* #1 and sh-*FMR1-AS1* #2 lentiviruses.

**d** GSEA of NFκB associated gene signatures in *FMR1-AS1* upregulated and downregulated cells, compared to respective control cells.

**e** Luciferase reporter assay in ECA-109 and KYSE-150 cells induced by TNF-α with or without NF-kB inhibition by sc-3060 or JSH-23, and the reporter constructs expressing the luciferase gene under *MYC* gene promoter segment (mean±SD, n=6, **p*<0.05, versus DMSO).

**f, e** *MYC* expression pattern in ESCC patients from TCGA and samples (n=48, ****P*<0.001)

**Figure s5.**

**a** qRT-PCR analysis of *FMR1-AS1* in the CM of ECA-109 and KYSE-150 cells treated with RNase (2 mg/ml) alone or combined with Triton X-100 (0.1%) for 20 min (n=3, **p*<0.05).

**b** *FMR1-AS1* expression in human blood exosomes obtained from exoRbase.

**c** NFκB activity of ESCC cells after 48 hr incubation with indicated exosomes, examined by luciferase reporter assay (n=3, **p*<0.05).

**d** Expression levels of c-Myc in ESCC cells after 48 hr incubation with indicated exosomes

**e** TOP-FLASH reporter assay in ESCC cells after 48 hr incubation with indicated exosomes.

**f** Western blotting showing active β-catenin, c-Jun, NICD and HES-1 in ESCC cells after 48 hr incubation with indicated exosomes.

**Figure s6.**

**a** Expression levels of c-Myc in ESCC cells with TLR7 knockdown after 48 hr incubation with indicated exosomes.

**b** Expression levels of c-Myc in ESCC cells with MyD88 knockdown after 48 hr incubation with indicated exosomes.
